# Single-cell transcriptional signature-based drug repurposing and in vitro evaluation in colorectal cancer

**DOI:** 10.1186/s12885-024-12142-8

**Published:** 2024-03-25

**Authors:** Roohallah Mahdi-Esferizi, Zahra Shiasi, Razieh Heidari, Ali Najafi, Issa Mahmoudi, Fatemeh Elahian, Shahram Tahmasebian

**Affiliations:** 1 Department of Medical Biotechnology, School of Advanced Technologies, Shahrekord University of Medical Sciences, Shahrekord, Iran; 2 Molecular Biology Research Center, Systems Biology and Poisonings Institute, Baqiyatallah University of Medical Sciences, Tehran, Iran; 3 Information Technology Department, Shahrekord University of Medical Sciences, Shahrekord, Iran; 4 Cellular and Molecular Research Center, Basic Health Sciences Institute, Shahrekord University of Medical Sciences, Shahrekord, Iran

**Keywords:** Drug repurposing, Transcriptional signature, Single-cell sequencing, iLINCS (Integrative LINCS), Colorectal cancer

## Abstract

**Background:**

The need for intelligent and effective treatment of diseases and the increase in drug design costs have raised drug repurposing as one of the effective strategies in biomedicine. There are various computational methods for drug repurposing, one of which is using transcription signatures, especially single-cell RNA sequencing (scRNA-seq) data, which show us a clear and comprehensive view of the inside of the cell to compare the state of disease and health.

**Methods:**

In this study, we used 91,103 scRNA-seq samples from 29 patients with colorectal cancer (GSE144735 and GSE132465). First, differential gene expression (DGE) analysis was done using the ASAP website. Then we reached a list of drugs that can reverse the gene signature pattern from cancer to normal using the iLINCS website. Further, by searching various databases and articles, we found 12 drugs that have FDA approval, and so far, no one has reported them as a drug in the treatment of any cancer. Then, to evaluate the cytotoxicity and performance of these drugs, the MTT assay and real-time PCR were performed on two colorectal cancer cell lines (HT29 and HCT116).

**Results:**

According to our approach, 12 drugs were suggested for the treatment of colorectal cancer. Four drugs were selected for biological evaluation. The results of the cytotoxicity analysis of these drugs are as follows: tezacaftor (IC10 = 19 µM for HCT-116 and IC10 = 2 µM for HT-29), fenticonazole (IC10 = 17 µM for HCT-116 and IC10 = 7 µM for HT-29), bempedoic acid (IC10 = 78 µM for HCT-116 and IC10 = 65 µM for HT-29), and famciclovir (IC10 = 422 µM for HCT-116 and IC10 = 959 µM for HT-29).

**Conclusions:**

Cost, time, and effectiveness are the main challenges in finding new drugs for diseases. Computational approaches such as transcriptional signature-based drug repurposing methods open new horizons to solve these challenges. In this study, tezacaftor, fenticonazole, and bempedoic acid can be introduced as promising drug candidates for the treatment of colorectal cancer. These drugs were evaluated in silico and in vitro, but it is necessary to evaluate them in vivo.

**Supplementary Information:**

The online version contains supplementary material available at 10.1186/s12885-024-12142-8.

## Introduction

Cancer is the second leading cause of death in the world, and colorectal cancer ranks fourth in incidence and third in mortality in the world [1]. Like other cancers, colorectal cancer cells act very smart and complex because they live in a microenvironment where cancer behavior results from the interaction of several factors, including immune cells, cancer cells, connective tissue cells and etc. Due to the heterogeneity of this cancer and to better understand this complexity, in 2015, a new classification system was presented in colorectal cancer called consensus molecular subtypes (CMSs). This system is robust because it includes various biological features such as transcriptome, mutation, copy number, methylation, microRNA, proteomics, and clinical data. Based on molecular characteristics, there are four classes: CMS1 (microsatellite instability immune), CMS2 (canonical), CMS3 (metabolic), and CMS4 (mesenchymal). From a clinical point of view, this system has potential in the field of diagnosis and treatment [2].

Cancer treatment is challenging and complicated, so demand for new effective drugs has increased. Drug discovery is very time-consuming (10–15 years) and expensive. Pharmaceutical Research and Manufacturers of America (PhRMA) invested $102.3 billion in 2021 in drug development, and the cost of drug discovery has a growing trend. Also, its efficiency could be higher; out of many drug candidates, only a few have been approved (2.01% on average). Therefore, the more innovative approach is drug repositioning (drug repurposing) instead of drug discovery. Drug repurposing means finding new indications for a drug that has already been approved by regulatory agencies (such as FDA) for a specific disease. For drug repurposing, some research is based on computational approaches, and others are based on biological approaches. But the best approach is to combine two approaches [3]. With the expansion of computational methods and biological data processing, various aspects of medicine, including predicting, diagnosing, and treating diseases, have made significant progress. Multiple approaches, including transcriptional signatures, network-based computational biology, molecular docking, structure-based methods, ligand-based chemogenomics, and machine learning, are used by researchers for drug repurposing [4].

An essential step in treating the disease is to have a comprehensive landscape of the molecular processes inside the cell and the interaction of cells with other cells and factors in their microenvironment. From the perspective of systems biology, a cell as a whole has several layers of different biological information that interact with each other. These biological data are known as omics data, which include: genomics, epigenomics, transcriptomics, proteomics, metabolomics, etc. Using High-Throughput Omics Technologies, a large amount of biological data has been generated. These data have revolutionized our understanding of the molecular processes in the cell. One of the goals of computational biology is to achieve multi-omics models that integrate all these biological data and give a single picture of the cell [5]. Currently, in the absence of such advanced models of the cell, transcriptomics data are helpful because they represent a vast and dynamic landscape of the molecular processes inside the cell. Three types of transcriptomic data are available. Microarray, bulk RNA seq, and single-cell RNA seq [6].

Every tissue consists of different types of cells; each type has a unique gene expression pattern. In the microenvironment of different tissues, each type of cell has a unique function in interaction with other cells that are genetically heterogeneous. Traditional transcriptomic methods, such as microarray and bulk RNA seq, represent the average gene expression of all cells in the tissue, so they lose part of the diverse genetic information, especially the types of cells that are few in the tissue but may be essential and effective. The advantage of single-cell sequencing technologies is that the sequencing of each cell is done separately so that it can consider the effect of each type of cell on the behavior of the tissue. By having a clear and high-resolution image of the role of each type of cell in the tissue opera, the understanding of molecular functions in the disease process will become closer to reality, and the methods of diagnosis and treatment of diseases will be more accessible [7].

**Fig. 1:**
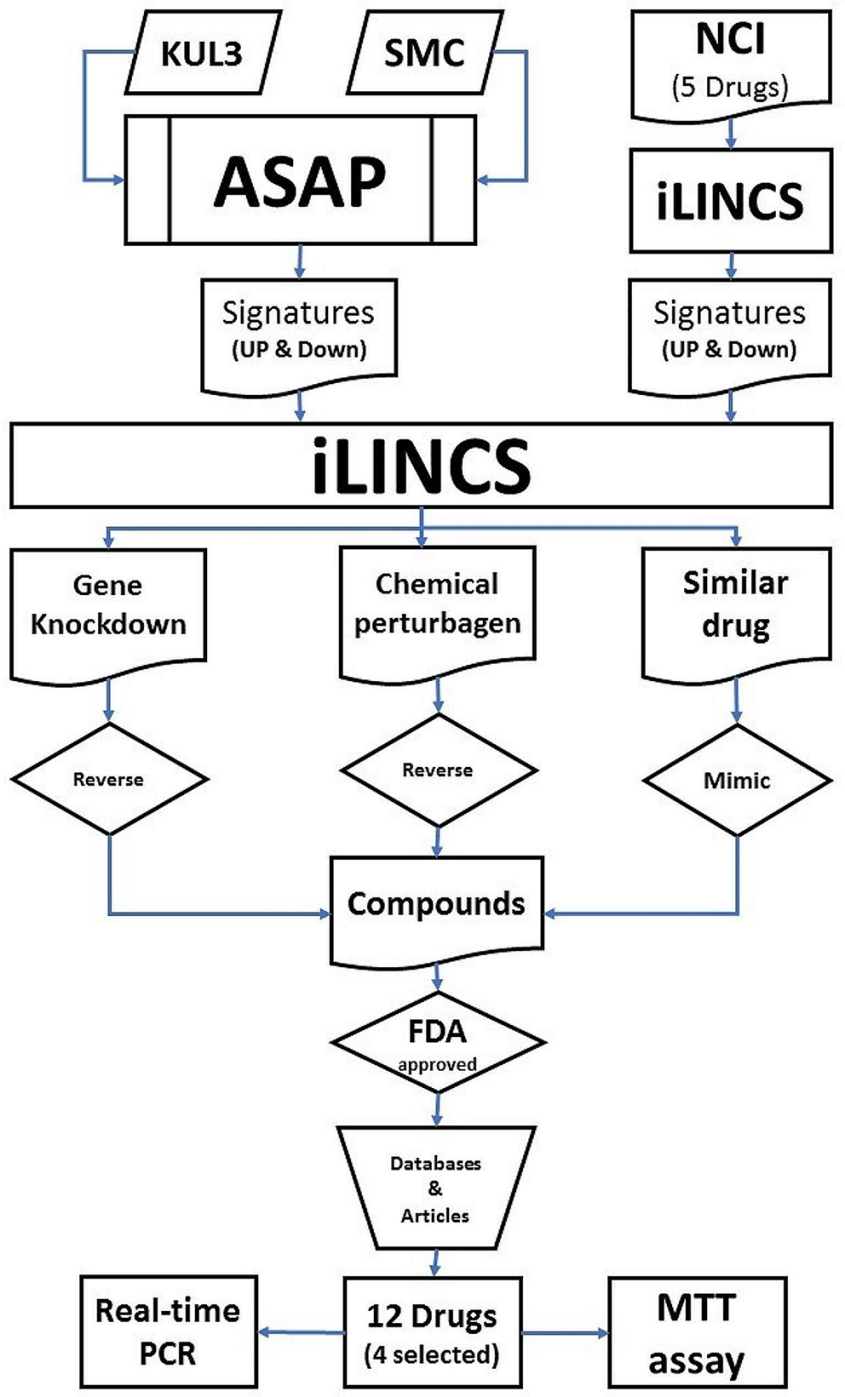
Workflow of our article: In the first step, two datasets (SMC & KUL3) were selected, and the gene signatures of the disease were calculated by the ASAP platform. Also, five common drugs for the treatment of colorectal cancer were selected from the NCI website, and their gene signatures were obtained using the iLINCS database.Then, with three different approaches, these gene signatures were checked by the iLINCS database, and chemical compounds that have the potential to treat colorectal cancer were selected. These compounds were filtered using various articles and pharmaceutical databases, and finally, 12 drugs were selected that had two conditions: being approved by institutions such as the FDA and not being introduced as a cancer treatment in any study so far. Four drugs were selected, and their cytotoxic effect on cancer cell lines (HT29 and HCT116) was evaluated by MTT assay and real-time PCR.

Two essential components are needed for drug repurposing with the transcriptional signature method: first, biologically valid datasets of gene signatures related to different drugs, and second, algorithms and platforms to process these data. The integrative Library of Integrated Network-Based Cellular Signatures (iLINCS) is a comprehensive web-based platform with various tools for visualization and analysis of omics data and transcriptional signatures [8]. iLINCS has 227,578 gene signatures related to about 15,000 chemical or genetic treatments in various diseases. More than one billion meaningful correlations have been found between these signatures, so with various tools, it is a robust database for drug discovery and drug repurposing. One of the functions of this platform is that by giving a gene signature related to any disease, it identifies chemical compounds or genetic interventions that can mimic or reverse the pattern of that gene signature. Therefore, it is possible to achieve compounds or genes that reverse the gene expression pattern of a disease such as cancer towards the normal state or mimic the gene expression pattern of a stem cell to a functional cell (differentiation process) [9]. In this study, using single-cell RNAseq data analysis, the gene signatures of colorectal cancer were obtained, and then 12 drugs were selected with three parallel approaches. Finally, the effect of cytotoxicity on four drugs was evaluated by the MTT assay, and using real-time PCR analysis, the expression of several genes was investigated. The flowchart of our study is shown in Fig. 1.

## Methods and materials

### Materials

The human colorectal cancer HCT-116 and HT-29 cell lines were purchased from the Pasteur Institute Cell Bank of Iran. Drugs (famciclovir, fenticonazole, tezacaftor, and Bempedoic acid) were purchased from MedChem Express. Total RNA extraction mini kit (Favorgen Biotech), SYBR®Green PCR Universal Mastermix and cDNA Synthesis Kit were ordered from Yekta Tajhiz Azma.

### Datasets

Two published scRNA-seq datasets in colorectal cancer were collected. The first scRNA-seq data was from a Korean population (GEO accession number GSE132465). The second scRNA-seq data was from a Belgian population (GEO accession number GSE144735). The producers of these datasets named the Korean population dataset “SMC” and the Belgian population dataset " KUL3” [10].

### Single cell analyses: finding signatures

SMC and KUL3 were analyzed separately but with the same pipeline and parameters using Automated Single-cell Analysis Pipeline (ASAP) Release 5: 2020-02-12 (https://asap.epfl.ch/). First, the expression matrices in raw_UMI_count format were uploaded to ASAP along with their annotation files. The annotation file contains information on the class (normal or tumor), type (epithelial, mast cell, etc.), and subtype of each cell. Then cell filtering was done. Cells with one of these conditions were excluded: less than 1000 UMI/reads, less than 100 detected genes, less than 80% protein-coding, more than 20% mitochondrial genes, and more than 40% ribosomal genes. The data were normalized using Seurat Normalization (ASAP implementation) method. Differential Expression (DE or DEA) analysis was performed using the Wilcoxon [Seurat] method. As mentioned before, these datasets had six types of cells. In this studied, the focus was on epithelial cells that were transformed into cancer cells. Epithelial cells had seven subtypes (Goblet cells, Intermediate, Mature Enterocytes type1 (MET1), Mature Enterocytes type2 (MET2), Stem-like/TA, Tuft, and Best 4 + enterocytes) in normal state and four different classes (CMS1, CMS2, CMS3, and CMS4) in cancer state. DE analysis was performed separately between these subtypes (as the normal group) and each of the four CMS classes (as the tumor group). The output genes of DE analysis with FDR (False Discovery Rate) less than 0.05 were selected as significant genes, and other genes were excluded. Then genes with 1 ≤ log fold change (LFC) ≤ -1 were considered as DEGs (LFC ≥ 1 as UP gene, and LFC ≤ -1 as DOWN gene). DEGs were considered as disease gene signatures. These gene signatures were used for enrichment analysis and finding drugs. Gene enrichment analysis (GEA) was performed to check the compatibility of gene signatures with biological research and better understand the molecular mechanisms and metabolic pathways involved in the disease. gene signatures were analyzed using the Enrichment Analysis Visualization Appyter, v0.2.6, Thu Apr 21 2022 (https://appyters.maayanlab.cloud/#/). Enrichment results with a P-value less than 0.05 were selected as significant.

### iLINCS: finding drugs

For each comparison that had made by performing DE analysis between each of the subtypes of epithelial cells with four CMS classes (for example, between mature enterocytes and CMS1 or mature enterocytes and CMS2, etc.), a gene signature file was prepared, which included the list of up and down genes and their LFC values. The iLINCS platform, v.2.8.0 (http://www.ilincs.org/ilincs/) analyzed the signature files, and the connected perturbations were obtained. Connected perturbations included two files: Connected LINCS gene knockdowns and Connected LINCS chemical perturbagens. The central concept in both files was a correlation, which was positive (mimic) or negative (reverse). In this database, a gene signature is created in the cell when a specific gene knocked down or overexpressed in a specific cell or treated with a specific chemical compound. These interventions (gene knockdown, gene overexpression, or chemical compound) were called perturbagens. The gene signature pattern of perturbagens was similar to the uploaded gene signature in positive correlation and its reverse in negative correlation. Three approaches were used in parallel to find the drugs:

***(a) Connected LINCS chemical perturbagens***: Finding compounds that could reverse the gene signature pattern of the disease (negative correlation). ***(b) Connected LINCS gene knockdowns***: It was found genes that reverse the gene signature pattern when knocked down. Then, instead of directly knocking down a gene with RNA interference (RNAi) or CRISPRs, inhibit the protein of the target gene with drugs. ***(c) Similar drugs***: The list of FDA-approved drugs for treating colorectal cancer was extracted from the National Cancer Institute (NCI) website (https://www.cancer.gov/), and the drugs that had gene signatures for large intestine tissue or colorectal cancer cell lines in the iLINCS database were selected. The signatures of these drugs were analyzed, and the compounds that could mimic these gene signatures (positive correlation) were selected. Therefore, the effect of these drugs on the cells is similar to the effect of the main drugs.

Finally, the compounds obtained from these approaches, which had the following two criteria, were selected. The rest were excluded: First, they must had approval from international organizations (such as The United States Food and Drug Administration (USFDA), Pharmaceuticals and Medical Devices Agency (PMDA), etc.) and had successfully passed the clinical trial stages. Secondly, these compounds had not been used in any cancer (either colorectal cancer or other cancer), so this was the first time these drugs had been used in cancer treatment. Each candidate compound was investigated by searching online articles and pharmaceutical databases such as ClinicalTrials (https://www.clinicaltrials.gov/), Drug Bank (https://go.drugbank.com/), NCATS Inxight Drugs (https://drugs.ncats.io/), PHAROS (https://pharos.nih.gov/), etc.

### Biological evaluation

After selecting drugs using three different approaches, two methods were used to evaluate their biological function on colorectal cell lines: cytotoxicity assay with MTT analysis and expression assay of some essential genes with real-time analysis. ***(a) cytotoxicity assay***: The human colorectal cancer HCT-116 and HT-29 cell lines were cultured in RPMI1640 medium supplemented with 10% fetal bovine serum (FBS), 100 unit/ml penicillin, and 100 µg/ml streptomycin at 95% humidity, 37 °C, and 5% CO2. Cell viability was evaluated using MTT (3-(4,5-dimethyl-2-thiazolyl)-2,5-diphenyl-2-H-tetrazolium bromide) assay. The logarithmic growth phase cells of HCT-116 and HT-29 cell lines at a density of 2*10^3 per well were seeded in 96-well plates and incubated for 24 h to allow attachment to the plate. Cells intervened with various concentrations of famciclovir, fenticonazole, tezacaftor, and bempedoic acid (100–1400 µM) of drugs diluted with cell culture media. Following a 48 h incubation period, 20 µl of MTT solution was added to each well and then incubated at 37 °C for 3 h. The medium was removed, and formazan was dissolved in 100 µL of dimethyl sulfoxide (DMSO). After gentle shaking, the optical density (OD) was measured at 570 nm using an ELISA reader. Notably, cells not exposed to drugs in the culture medium were considered the control, and each concentration’s viability was assessed compared to the control. The mean cell viability (%) was plotted against the compound’s concentration (µM) to create dose-response curves and inhibitory concentration 10 (IC10) values were then determined by linear regression in Microsoft Excel. ***(b)Real time-PCR***: To evaluate the effects of each drug on the cell, two genes were selected to be analyzed by the real-time method: an up gene and a down gene. Up gene was expected to have an increased expression in the cell treated with the drug. To find them, two lists were prepared: the list of down genes in the disease signature (obtained from ASAP) and the list of up genes in the signature of the drug (obtained from iLINCS). Then, using the Venn diagram (https://bioinfogp.cnb.csic.es/tools/venny/), the intersecting genes of the two lists were determined. Down gene was expected to have a decrease expression in the cell treated with the drug. The same process was done to find the down genes. Therefore, common genes were determined between two lists (one list of genes with high expression in disease and one list of genes with low expression in drug treatment). This method was used to find genes in famciclovir, fenticonazole and tezacaftor. There was no gene signature for bempedoic acid in the iLINCS database, so articles were used to select the appropriate gene for real-time analysis.

Total RNA samples of HCT-116 and HT-29 cells were extracted by TRIzol® reagent. RNA was qualified and quantified with the nanodrop spectrophotometer instrument. Then, 1000 ng of RNA extracted from the sample was used for cDNA synthesis according to the manufacturers’ manuals of the Yekta Tajhiz Kit. Quantitative Real time-PCR was performed using SYBR®Green PCR Universal Mastermix of Yekta Tajhiz kit. Thermocycler conditions included initial denaturation at 95 °C for 10 min, followed by 40 cycles of denaturation at 95 °C for 15 s, annealing at 60 °C for 30 s, and extension at 72 °C for 30 s. Primers were designed with gene runner software. The changes in the RNA expression in all the groups were calculated by the Pfaffl method using β-actin as an endogenous reference to normalize the gene expression levels in each sample. LinReg PCR 2013 software was used to calculate the amplification efficiency of each of the primers. It should be noted that amplifications of samples, standards, and controls were run in triplicate in the Rotor-Gene Q device. One-way analysis in GraphPad Prism was used to calculate statistical significance using all experimental values. The results of two independent experiments in triplicates are represented as the mean ± standard deviation (SD). A P-value ≤ 0.05 was considered to denote a statistically significant difference.

## Results

### Signatures and Enrichment

Two scRNA-seq datasets were analyzed. SMC contained 63,689 cells (16,404 normal cells and 47,285 tumor cells) from 23 patients. KUL3 consisted of 27,414 cells (9736 normal cells, 9424 border cells, and 8254 tumor cells) from 6 patients. Both datasets represented 33,694 genes from six cell types (Epithelial, Stromal, Myeloid, T, B, and Mast cells). The number and types of cells and their subtypes in each dataset were shown in Table S1 in Additional file 1. The result of DE analysis was a list of genes with their FDR and LFC values. If the LFC value of a gene was equal to 1 (up gene), the expression of this gene in the tumor state was twice the normal state (2^1 = 2), and if the LFC value was equal to -1 (down gene), its expression in the tumor state was half of the normal state (2^(-1) = 0.5). The number of up and down genes (gene signature) in each DE analysis was shown in Table S2 in Additional file 1. Four types of enrichment (CCLE, GO_BP, ChEA, WikiPathway) were performed on gene signatures using Appyter, and enrichment results were shown in Table S3 in Additional file 1. The files of DEGs and Enrichment results are available in Additional file 2_Part 1.

### iLINCS: finding drugs

**Fig. 2:**
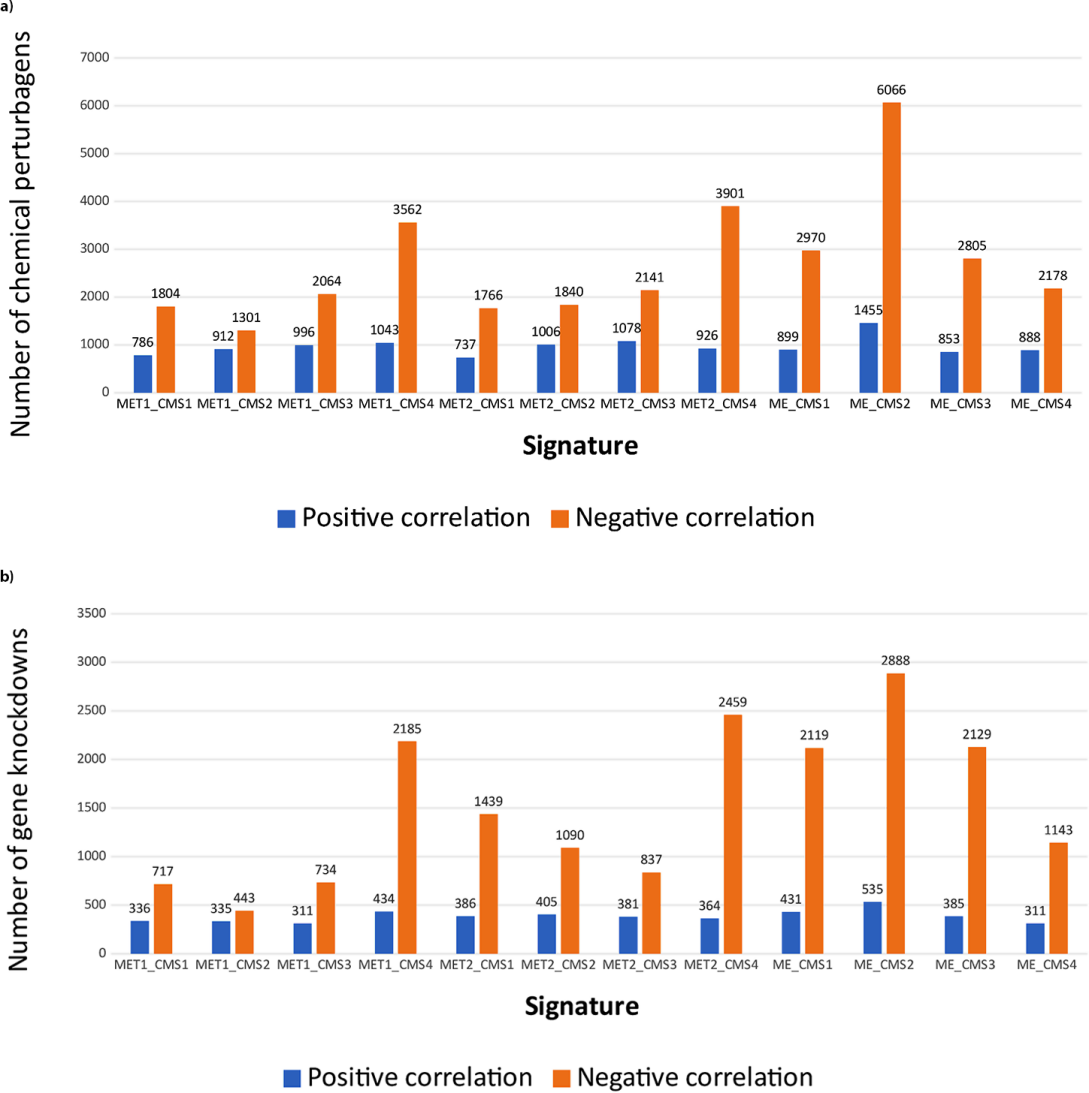
The results obtained from the iLINCS using gene signature data. The number of chemicals perturbagens (**a**) and gene knockdowns (**b**) in positive (mimic) and negative (reverse) correlation

Three approaches were used to find the drug: The first approach was to select chemical molecules that could reverse the disease gene signature. Figure 2 (a) showed the number of chemical molecules found in mimic and reverse correlation for each CMS (MET1 and MET2 respectively represented Mature Enterocyte type 1 and Mature Enterocyte type 2 in the SMC dataset, and ME represented Mature Enterocyte in the KUL3 dataset). The output file of the iLINCS website contained the name and ID of the chemical compounds, target genes of the compound, correlation, number of gene signatures, P-value and Z-score. In each file, 50 compounds with the highest z-score were selected from the reverse states (negative correlation) for further investigation. The second approach was to select genes that, if knocked down, reverse the gene signature of the disease. Figure 2 (b) showed the number of genes found in both mimic and reverse correlation for each CMS. The output file of the iLINCS website contained the target gene name, pathway, correlation, number of gene signatures, P-value, and Z-score. The genes with the highest z-score were selected from the reverse states (negative correlation) in each file. Then, drugs that inhibited these genes’ proteins were obtained using the database. The third approach was to select drugs whose gene signature in colorectal cancer was similar to those approved by the FDA in treating colorectal cancer. Five drugs were selected: Irinotecan Hydrochloride, Fluorouracil, Trifluridine and Tipiracil Hydrochloride, Regorafenib, and Floxuridine. Finally, 12 drugs were obtained from three approaches, as shown in Table 1. The files of connected LINCS chemical perturbagens and connected LINCS gene knockdowns for each dataset and similar drugs are available in Additional file 2_Part 2 and Part 3. Details of the four drugs selected for biological evaluation and their target genes and related compounds are available in Table S5, S6, and S7 in Additional file 1. (The data was extracted from the iLINCS website).

**Table 1:** The list of drugs obtained from the three approaches of chemical perturbagens, gene knockdowns and similar drugs

Chemical perturbagens		Gene knockdowns			Similar drugs
CMS	Drug	CMS	Gene	Drug	Drug
CMS3	Famciclovir	CMS1	HSPD1	DiaPep277	Fenticonazole Nitrate
CMS4	Gestrinone	CMS2	CFTR	tezacaftor	Diphenylpyraline
	Ecabet		ABAT	vigabatrin	Tezampanel
	Y-39,983	CMS4	ACLY	Bempedoic acid	BMY-14,802

### Biological evaluation: MTT assay & real time-PCR

Cell viability of HCT-116 and HT-29 cell lines was measured after exposure to various concentrations of Famciclovir, Fenticonazole, Tezacaftor, and Bempedoic acid for 48 h. The IC10 value of Famciclovir, Fenticonazole, Tezacaftor, and Bempedoic acid after 48 h of treatment was shown in Table 2. Based on the MTT results, Bempedoic acid and Fenticonazole presented higher toxicity than Famciclovir and Tezacaftor in HCT-116, which caused a significant reduction in cell viability. Furthermore, Tezacaftor and Fenticonazole displayed significant anti-tumor activity compared to Bempedoic acid and Famciclovir in HT-29 cells, which inhibited cell viability in a dose and time dependent manner.

**Table 2:** Inhibitory concentration 10 (IC10) of repurposed drugs in HCT-116 and HT-29 cell line. The unit of IC10 was in micromolar (µM)

Cell line/Drug	Famciclovir	Fenticonazole	Tezacaftor	Bempedoic acid
HCT-116	422	17	19	78
HT-29	959	7	2	65

For real-time analysis, up and down genes were selected whose expression patterns were opposite in the disease state compared to the drug treatment state (Table S4 in Additional file 1). To determine the effects of Famciclovir, Fenticonazole, Tezacaftor, and Bempedoic acid on cancer progression, the genes related to tumor progression MMP1, BAD, STMN1, SFN, SOX4, Cdh1, and Twist1 were selected for investigation by Real time-PCR method (Table 3).

**Table 3:** Genes analyzed in each drug and their primer sequences, as well as the primer sequence of beta-actin gene as a control gene (endogenous reference)

Drugs	Genes	Primer sequence
Bempedoic acid	Cdh1	Forward: 5´- GCTGTTTCTTCGGAGGAGAGCG-3´ Reverse: 5´- CATGAGGGTTGGTGCAACGTCG-3´
	Twist-1	Forward: 5´-GGAGTCCGCAGTCTTACGAGG-3´ Reverse: 5´-GCTCTGGAGGACCTGGTAGAG-3´
Famciclovir	MMP1	Forward: 5´-GGGAAACCAGATGCTGAAACC-3´ Reverse: 5´- GGCTTTCTCAATGGCATGGTCC-3´
Tezacaftor	SFN	Forward: 5´- TCATTGACTCAGCCCGGTCAGC-3´ Reverse: 5´- TGTCAGGTTGTCTCGCAGCAGC-3´
	SOX4	Forward: 5´- AACCAACAATGCCGAGAACACG-3´ Reverse: 5´- ATCTGCGACCACACCATGAAGG-3´
Fenticonazole	BAD	Forward: 5´- TCCTGGTGGGATCGGAACTTGG-3´ Reverse: 5´- TCACACGCACCGGAAGGGAATC-3´
	STMN1	Forward: 5´- AACTGGAGAAGCGTGCCTCAGG-3´ Reverse: 5´- TCAGCTTCATGGGACTTGCGTC-3´
Endogenous reference	β-actin	Forward: 5′ -TCATGAAGTGTGACGTGGACATC 3′ Reverse: 5′ CAGGAGGAGCAATGATCTTGATCT 3′

**Fig. 3:**
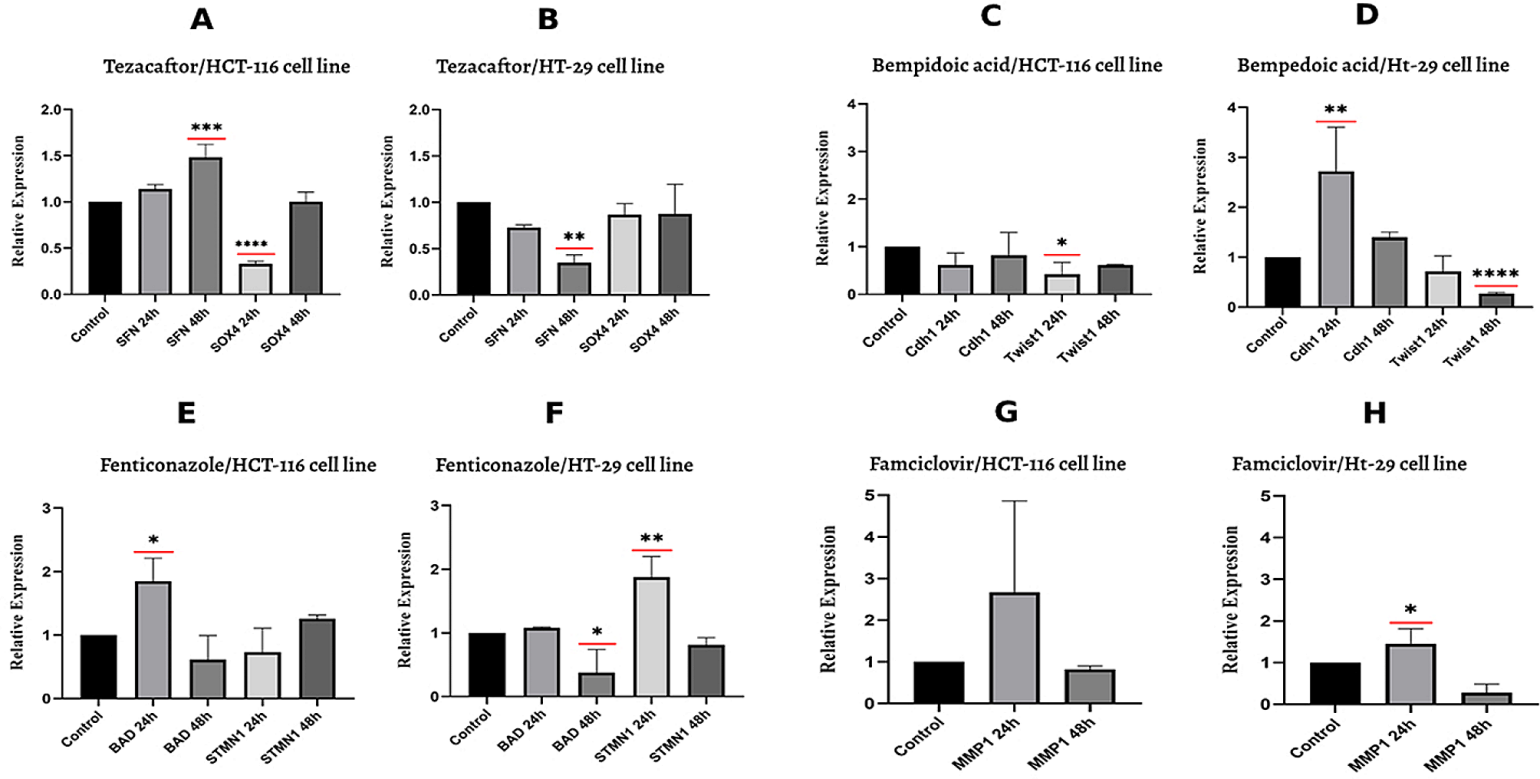
The results of real-time PCR in HCT-116 and HT-29 cell lines after 24 and 48 h of drug treatment. SFN and SOX4 expression tezacaftor treatment on the HCT-116 (**A**) and HT-29 (**B**). Cdh1 and Twist1 expression of bempedoic acid treatment on the HCT-116 (**C**) and HT-29 (**D**). BAD and STMN1 expression of Fenticonazole treatment on the HCT-116 (**E**) and HT-29 (**F**). MMP1 expression of famciclovir treatment on the HCT-116 (**G**) and HT-29 (**H**). *, ** and ***Symbol denotes significance at *P* < 0.05, 0.01 and 0.001 levels

Cells were treated with IC10 concentration of drugs at 24 and 48 h after treatment, and then, real-time-PCR measured gene expression. The results demonstrated that tezacaftor treatment of HCT-116 cells led to a significant increase in SFN expression after 48 h and a significant decrease in SOX4 expression after 24 h (Fig. 3 (A)). Tezacaftor treatment of HT-29 cells significantly decreased SFN expression after 48 h (Fig. 3 (B)). Bempedoic acid treatment of HCT-116 cells led to a significant decrease in Twist1 gene expression after 24 h (Fig. 3 (C)). Furthermore, Bempedoic acid treatment of HT-29 cells significantly decreased Twist1 expression after 48 h and increased Cdh1 expression after 24 h (Fig. 3 (D)). The results revealed that fenticonazole treatment of HCT-116 cells caused significantly increased level expression of BAD after 24 h (Fig. 3 (E)). By contrast, fenticonazole treatment of HT-29 cells is associated with significantly decreased expression of BAD after 48 h and increased STMN1 after 24 h (Fig. 3 (F)). Famciclovir treatment of HCT-116 cells showed non-significant MMP1 expression alteration after 24 and 48 h (Fig. 3 (G)), while famciclovir treatment of HT-29 cells led to significantly decreased MMP1 expression level after 48 h (Fig. 3 (H)).

## Discussion

Colorectal cancer was identified as the second- to fourth-most common type of cancer worldwide. Colorectal cancer was expected to record significant mortality among digestive tract cancers because of genetic and environmental factors, including age, gender, race, obesity, physical inactivity, smoking, alcohol consumption, and inappropriate dietary habits. The computational drug repositioning approach offered a time- and cost-efficient way to expand treatment options for oncologic patients via established FDA-approved or investigational candidate drugs. Anselmino explored the effect of combining the repositioned drugs metformin and propranolol in both colorectal and triple-negative breast cancers, finding that the combination could be useful as a putative adjuvant treatment for both types of cancer [11]. Yong demonstrated that treatment with Linagliptin of HCT116 cells prevented the growth of colorectal cancer tumor cells, causing cell cycle arrest and inducing apoptosis [12]. Beklen used differential interactome-based drug repositioning to identify potential drug candidates for CRC treatment, including abacavir, exemestane, nortriptyline hydrochloride, and tolcapone [13]. Fong reviews the preclinical and clinical efficacy of repurposed drugs, including non-steroidal anti-inflammatory drugs, statins, metformin, chloroquine, disulfiram, niclosamide, zoledronic acid, and angiotensin receptor blockers [14]. Noort used drug-induced gene-expression profiles to predict novel drugs for colorectal cancer, including citalopram, troglitazone, and enilconazole [15]. This study applied drug repurposing strategies to colorectal cancer gene expression profiles to identify repurposable drugs that could potentially reverse the gene expression signatures and ultimately led to disease suppression. Bioinformatics datasets and academic articles were used in the initial step to screen certain prospective colorectal cancer drugs. The anti-tumor potential of a few drugs was then examined in the colorectal cancer cell lines HCT-116 and HT-29 to validate their effectiveness against colorectal cancer.

Tezacaftor is a drug commonly used to treat cystic fibrosis (CF). Tezacaftor functions as a cystic fibrosis transmembrane conductance regulator (CFTR) corrector that stabilizes the misfolded F508del mutation of the CFTR gene [16]. Increasing evidence demonstrated the close association between inactivating mutations in the CFTR and susceptibility to colorectal tumor development [17]. Low CFTR expression in colorectal cancer probably results from silencing the CFTR gene by promoter hypermethylation [18]. Notably, drugs targeting CFTR may find application in colorectal cancer. Tezacaftor may have potent anticancer activity against colorectal cancer and improve metastatic processes through its impact on the CFTR gene, consistent with this study. Tétard et al. demonstrated that Lumacaftor/Ivacaftor, CFTR modulators, could reduce intestinal inflammation in CF patients [19]. Our bioinformatics results proposed that 13 genes (IGFBP3, S100A13, MMP1, CDKN2A, MYL9, SOX4, FKBP4, DUSP4, GLRX, UBE2L6, NFATC4, NUCB2) were upregulated, whereas three genes (SFN, IER3, ALDOA) were downregulated in colorectal cancer (Table S4 in Additional File 1). Further analysis showed these genes were relevant to cell differentiation, apoptosis, and malignancies, which were traceable in the literature. Our quantitative PCR results demonstrated that tezacaftor might promote SFN gene expression in HCT-116 cells, preventing colorectal cancer progression. Although SFN was overexpressed in HCT-116 cells, its expression in HT-29 cells was decreased. Controversial studies reported the role of SFN in potentiating colon tumorigenesis through the activation of various factors, such as matrix metalloproteinase 28 (MMP28), which contributed to the progression of colorectal cancer. Therefore, tezacaftor probably repressed the expression of the SFN gene and led to colorectal cancer progression, which supported our results in HT-29. Further studies would clarify the effects of the increased expression of SOX4 with tumorigenesis and progression in colorectal cancer [20, 21]. Our results verified that the expression of SOX4 was significantly suppressed by tezacaftor in HCT-116 cells. In summary, consistent with our hypothesis, CFTR modulator drugs such as Tezacaftor, with great promise for performance, may be repurposed for use in treating CFTR-deficient colorectal cancer patients in both CF and non-CF patients.

Bempedoic acid inhibits lipid synthesis by suppressing ATP citrate lyase, a key enzyme in the cholesterol biosynthesis pathway. The correlation between cholesterol measurement and colorectal cancer was less well known. Although the inverse association was seen in most previous studies [22], supporting evidence reported that high cholesterol levels were a well-established risk factor for colorectal cancer, and bempedoic acid might have anti-colorectal effects by suppressing cholesterol biosynthesis [23]. Based on our studies, inhibiting lipid synthase would be a promising strategy to treat colorectal cancer tumors. Accordingly, Kirdel et al. showed that orlistat, an inhibitor of fatty acid synthase, had a cytotoxic effect on human colorectal cancer by inducing apoptosis in the human colon cancer [24]. Twist1 participates in chromosomal and genomic instability and downregulates critical cell cycle checkpoint factors in colorectal cancer cells [25]. It was reported that Twist1 was involved in the metastasis of cancers that could induce EMT [26]. Furthermore, Twist1 was a well-known repressor of the E-cadherin gene (CDH1) during EMT [27]. Additionally, in vitro evidence showed that increased CDH1 and decreased expression levels of Twist1 result from bempedoic acid treatment in HT-29. These results prompted the hypothesis that the upregulation of Twist1 and, subsequently, the downregulation of CDH1 might predispose human patients to colorectal cancer [28]. As expected, the observed decreases in Twist1 and increases in CDH1 were potentially related to Bempedoic acid influences.

Fenticonazole is an imidazole antifungal drug used to treat various topical fungal infections. Intriguingly, the studies provided evidence that Fenticonazole would have value in cancer therapy, whereas the underlying mechanism of the anti-cancer effect of fenticonazole on different kinds of tumors remained poorly understood [29, 30]. Shen et al. found that itraconazole, an antifungal drug, reduced the survival rate of colon cancer cells and downregulated genes associated with proliferation and migration [31]. Shi et al. provided evidence that oxiconazole, an antifungal drug, statistically significantly affected colorectal cancer risk. Indeed, oxiconazole induced apoptosis in human colon cancer cells and inhibited tumor progression [32]. Our bioinformatics approach identified patterns of gene expression that were linked to colorectal cancer. For example, the expression level of four genes, such as BAD, decreased in colorectal cancer, while the expression level of 28 genes, such as STMN1, increased in colorectal cancer (Table S4 in Additional File 1). The potential targets for Fenticonazole included BAD, which was involved in cell apoptosis, and it was demonstrated that the highest expression levels of BAD were closely correlated with tumor suppression [33]. We demonstrated that Fenticonazole could strongly induce the killing of colorectal cancer cells via a mechanism involving the suppression effect of proapoptosis BAD in HCT-116 [34]. An additional target identified by our approach was Stathmin 1 (STMN1); however, the result from qRT-PCR was contradictory. It had to be pointed out that inhibition of STMN1 led to cell cycle arrest in the G2/M phase and, subsequently, suppression of cellular proliferation. Our observation revealed that Fenticonazole therapy could not repress STMN1 expression in HT-29 [35]. Considering that the in vitro results of STMN1 and BAD in colorectal treatment were inconsistent, our results suggested that Fenticonazole may not provide a new therapeutic action in colorectal cancer and that it needed more research to be introduced as an anti-cancer drug.

Famciclovir exerted antiviral effects due to its ability to disrupt intracellular nucleic acid processing of varicella-zoster virus (VZV) and herpes simplex virus types 1 (HSV-1) and 2 (HSV-2). Although the association between Famciclovir and cancer remained obscure, some clues indicated that antiviral drugs suppressed colorectal cancer [36]. Multiple studies revealed that VZV, HSV-1, and HSV-2 showed significant prevalence in tumor-associated colorectal cancer [37]. In this respect, these findings suggested that it was not unreasonable to consider that Famciclovir exhibited anti-colorectal cancer activity in addition to its intrinsic direct-acting antiviral function [38]. These findings were consistent with previous reports by Zhou et al. It showed that Ivermectin, an anti-virus drug, decreased cell proliferation and enhanced its anti-cancer effect on human colorectal cancer [39]. Our computational study confirmed that colorectal cancer had also been associated with the upregulation of 16 genes, such as MMP1, and the downregulation of one gene (ACAA1). A gene selected for further investigation was MMP1. MMP1 had been previously identified as a promising therapeutic target in colorectal cancer. Our results supported this contention and indicated that Famciclovir could have an anti-cancer effect in colorectal cancer by inhibiting MMP1 expression in HT-29, according to qRT-PCR result [40]. These findings supported the therapeutic action of Famciclovir as a new drug in colorectal cancer.

## Conclusion

We showed that mining and developing in silico tools and in vitro analysis approaches based on cancer gene expression profiles and the effect of drugs on gene expression could open up a promising strategy for colorectal cancer treatment. Applying iLINCs to colorectal cancer data has allowed different research groups to discover many potential drugs to suppress colorectal cancer. It should be noted that the potential effect of Tezacaftor, bempedoic acid, famciclovir, and fenticonazole drugs in repressing colorectal cancer aggressiveness is still a hypothesis and needs to be monitored for the development of future treatment regimens in patients.

### Electronic supplementary material

Below is the link to the electronic supplementary material.

Supplementary Material 1

Supplementary Material 2

## Data Availability

The datasets generated or analyzed during the current study are available on request from the corresponding author.

## Abbreviations

ASAP: Automated Single-cell Analysis Pipeline
CF: cystic fibrosis
CFTR: cystic fibrosis transmembrane conductance regulator
CMS: consensus molecular subtypes
DEGs: Differentially Expressed Genes
DMSO: dimethyl sulfoxide
EMT: Epithelial-mesenchymal transition
FBS: fetal bovine serum
FDR: False Discovery Rate
GEA: Gene enrichment analysis
HSV-1: herpes simplex virus types 1
HSV-2: herpes simplex virus types 2
IC10: inhibitory concentration 10
iLINCS: integrative Library of Integrated Network-Based Cellular Signatures
LFC: log fold change
MET1: Mature Enterocytes type1
MET2: Mature Enterocytes type2
MMP28: matrix metalloproteinase 28
MTT: (3-(4,5-dimethyl-2-thiazolyl)-2,5-diphenyl-2-H-tetrazolium bromide)
NCI: National Cancer Institute
OD: optical density
STMN1: Stathmin 1
