# Supplementary material for: Single-cell transcriptional signature-based drug repurposing and in vitro evaluation in colorectal cancer
Source: BMC Cancer. 2024 Mar 25;24:371. doi: 10.1186/s12885-024-12142-8 (PMC10962075; doi:10.1186/s12885-024-12142-8)
Supplement: Supplementary file 1 — Supplementary Material 1 [file 12885_2024_12142_MOESM1_ESM.docx]

**Single-cell transcriptional signature-based drug repurposing and in vitro evaluation in colorectal cancer**

**Roohallah Mahdi-Esferizi^1^, Zahra Shiasi^1^,** **Razieh Heidari^1^, Ali Najafi^2^, Issa Mahmoudi^3^, Fatemeh Elahian^1^, and Shahram Tahmasebian^4,^***

^1^ Department of Medical Biotechnology, School of Advanced Technologies, Shahrekord University of Medical Sciences, Shahrekord, Iran.

^2^ Molecular Biology Research Center, Systems Biology and Poisonings Institute, Baqiyatallah University of Medical Sciences, Tehran, Iran.

^3^ Information Technology Department, Shahrekord University of Medical Sciences, Shahrekord, Iran.

^4^ Cellular and Molecular Research Center, Basic Health Sciences Institute, Shahrekord University of Medical Sciences, Shahrekord, Iran.

* Corresponding author

**Shahram Tahmasebian**

Cellular and Molecular Research Center, Basic Health Sciences Institute, Shahrekord University of Medical Sciences, Shahrekord, Iran ([stahmasebian@gmail.com](mailto:stahmasebian@gmail.com))

Table S1: The number and types of cells and their subtypes in the Korean population dataset "SMC" and the Belgian population dataset "KUL3".

| **Cell type** | **Cell subtype** | **Korean_Normal (16404)** | **Korean_Tumor (47285)** | **Belgian_Normal**  **(9736)** | **Belgian_Border**  **(9424)** | **Belgian_Tumor**  **(8254)** |
| --- | --- | --- | --- | --- | --- | --- |
| **B cells** | CD19+CD20+ B | 1684 | (2049) | 441 | 934 | 470 |
|  | IgA+ Plasma | 3366 | (180) | 2049 | 509 | 159 |
|  | IgG+ Plasma | 94 | (1661) | 4 | 17 | 84 |
|  | Unknown | 64 | (48) | 110 | 12 | 12 |
|  | Unspecified Plasma | - | - | - | 49 | 52 |
| **Epithelial cells** | Goblet cells | 139 | - | 165 | 2812 | - |
|  | Intermediate | 181 | - | 253 |  | - |
|  | Mature Enterocytes type 1 | 213 | - | 153 |  | - |
|  | Mature Enterocytes type 2 | 131 | - | - |  | - |
|  | Stem-like/TA | 406 | - | 495 |  | - |
|  | tuft | - | - | 26 |  | - |
|  | best 4+ enterocytes | - | - | 52 |  | - |
|  | CMS1 | - | (1201) | - |  | 758 |
|  | CMS2 | - | (10771) | - |  | 620 |
|  | CMS3 | - | (5486) | - |  | 804 |
|  | CMS4 | - | (11) | - |  | 30 |
| **Mast cells** | - | 184 | 3 | 62 | 108 | 78 |
| **Myeloids** | cDC | (129) | (353) | 60 | 51 | 51 |
|  | Pro-inflammatory | (74) | (2325) | 265 | 209 | 230 |
|  | Proliferating | (6) | (165) | 39 | 116 | 119 |
|  | SPP1+ | (156) | (3096) | - | - | - |
|  | SPP1+ A |  |  | 1 | 48 | 54 |
|  | SPP1+ B |  |  | 7 | 329 | 298 |
|  | Anti-inflammatory | - | - | 406 | 128 | 122 |
|  | Unknown | (4) | (461) | 45 | 43 | 55 |
| **Stromal cells** | Enteric glial cells | (282) | (23) | 391 | 41 | 31 |
|  | Lymphatic ECs | (80) | (28) | 66 | 20 | 37 |
|  | Myofibroblasts | (9) | (1146) | 26 | 422 | 778 |
|  | Pericytes | (92) | (353) | 72 | 165 | 201 |
|  | Smooth muscle cells | (123) | (91) | 273 | 126 | 192 |
|  | Stalk-like ECs | (97) | (342) | 139 | 159 | 225 |
|  | Stromal 1 | (972) | (73) | 417 | 116 | 159 |
|  | Stromal 2 | (236) | (158) | 151 | 37 | 9 |
|  | Stromal 3 | (744) | (124) | 1763 | 368 | 336 |
|  | Tip-like ECs | (563) | (354) | 266 | 311 | 353 |
|  | Proliferative ECs | - | (44) | - | - | - |
| **T cells** | CD4+ T cells | (3325) | (3980) | 609 | 798 | 654 |
|  | CD8+ T cells | (1757) | (4647) | 503 | 439 | 384 |
|  | gamma delta T cells | (777) | (219) | 138 | 15 | 6 |
|  | NK cells | (163) | (948) | 104 | 86 | 80 |
|  | Regulatory T cells | (154) | (2943) | 46 | 488 | 577 |
|  | T follicular helper cells | (72) | (548) | 21 | 153 | 85 |
|  | T helper 17 cells | (17) | (1961) | 116 | 165 | 31 |
|  | Unknown | (111) | (1493) | 2 | 150 | 120 |

Table_S 2: The number of up and down genes (gene signature) between each subtype and each CMS in two datasets (SMC & KUL3). All the results are shown here, but in the following we focus on signatures of Mature Enterocyte type 1 (MET1) and Mature Enterocyte type 2 (MET2) in the SMC dataset and Mature Enterocyte (ME) in the KUL3 dataset to find the drugs.

| datasets | State  Tumor vs Normal | | Signature (DEGs) | | State  Tumor vs Normal | | Signature (DEGs) | |
| --- | --- | --- | --- | --- | --- | --- | --- | --- |
|  | Normal | tumor | up | down | Normal | tumor | up | down |
| SMC | Goblet | CMS1 | 181 | 109 | MET1 | CMS1 | 417 | 162 |
|  | Goblet | CMS2 | 171 | 117 | MET1 | CMS2 | 358 | 186 |
|  | Goblet | CMS3 | 124 | 83 | MET1 | CMS3 | 293 | 162 |
|  | Goblet | CMS4 | 58 | 58 | MET1 | CMS4 | 264 | 117 |
|  | intermediate | CMS1 | 127 | 59 | MET2 | CMS1 | 429 | 178 |
|  | intermediate | CMS2 | 62 | 51 | MET2 | CMS2 | 347 | 191 |
|  | intermediate | CMS3 | 83 | 61 | MET2 | CMS3 | 302 | 178 |
|  | intermediate | CMS4 | 146 | 36 | MET2 | CMS4 | 275 | 100 |
|  | Stem-likeTA | CMS1 | 105 | 49 | Stem-likeTA | CMS3 | 63 | 42 |
|  | Stem-likeTA | CMS2 | 50 | 40 | Stem-likeTA | CMS4 | 49 | 216 |
| KUL3 | BEST 4+ Enterocytes | CMS1 | 106 | 122 | intermediate | CMS1 | 151 | 101 |
|  | BEST 4+ Enterocytes | CMS2 | 79 | 167 | Intermediate | CMS2 | 40 | 78 |
|  | BEST 4+ Enterocytes | CMS3 | 86 | 107 | Intermediate | CMS3 | 148 | 93 |
|  | BEST 4+ Enterocytes | CMS4 | 68 | 203 | intermediate | CMS4 | 129 | 188 |
|  | Goblet | CMS1 | 155 | 112 | ME | CMS1 | 190 | 191 |
|  | Goblet | CMS2 | 164 | 147 | ME | CMS2 | 168 | 236 |
|  | Goblet | CMS3 | 124 | 91 | ME | CMS3 | 159 | 170 |
|  | Goblet | CMS4 | 102 | 164 | ME | CMS4 | 171 | 300 |
|  | Stem-likeTA | CMS1 | 121 | 79 | Tuft | CMS1 | 292 | 124 |
|  | Stem-likeTA | CMS2 | 51 | 85 | Tuft | CMS2 | 259 | 224 |
|  | Stem-likeTA | CMS3 | 103 | 67 | Tuft | CMS3 | 246 | 113 |
|  | Stem-likeTA | CMS4 | 62 | 127 | Tuft | CMS4 | 96 | 125 |

Table_S 3: Enrichment results. Four types of enrichment were performed on gene signatures (between each subtype and each CMS in two datasets). CCLE (Cancer Cell Line Encyclopedia), GO_BP (Gene Ontology _Biological Process_2021), ChEA (ChIP Enrichment Analysis_2022), WikiPathway (WikiPathway_2021), Mature Enterocyte type 1 (MET1), Mature Enterocyte type 2 (MET2), Mature Enterocyte (ME).

| **Dataset** | **Normal** | **Cancer** | **Enrichment method** | **#** | **Examples** |
| --- | --- | --- | --- | --- | --- |
| **SMC** | MET1 | CMS1 | CCLE | 63 | CL34 LARGE INTESTINE  SNU283 LARGE INTESTINE  CL40 LARGE INTESTINE |
|  |  |  | GO_BP | 564 | epithelial structure maintenance (GO:0010669)  maintenance of gastrointestinal epithelium (GO:0030277)  epithelial cell differentiation (GO:0030855) |
|  |  |  | ChEA | 259 | MYC  XRN2 |
|  |  |  | WikiPathway | 52 | Metabolic reprogramming in colon cancer WP4290  VEGFA-VEGFR2 Signaling Pathway WP3888 |
|  |  | CMS2 | CCLE | 45 | CL34 LARGE INTESTINE  T84 LARGE INTESTINE  SNU283 LARGE INTESTINE |
|  |  |  | GO_BP | 517 | regulation of epithelial cell migration (GO:0010632)  mitotic nuclear membrane organization (GO:0101024)  regulation of epithelial cell proliferation (GO:0050678) |
|  |  |  | ChEA | 298 | MYC 19079543 ChIP-ChIP MESCs Mouse  MYC 18555785 ChIP-Seq MESCs Mouse  MYC 28411283 ChIP-Seq MDA231-LM2-4175 Human BreastCancer  MYC 19030024 ChIP-ChIP MESCs Mouse  MYC 18358816 ChIP-ChIP MESCs Mouse  XRN2 22483619 ChIP-Seq HELA Human  E2F1 18555785 ChIP-Seq MESCs Mouse  MYC 22102868 ChIP-Seq CA46 Human Blood BurkittsLymphoma  TTF2 22483619 ChIP-Seq HELA Human |
|  |  |  | WikiPathway | 68 | VEGFA-VEGFR2 Signaling Pathway WP3888  Metabolic reprogramming in colon cancer WP4290  Retinoblastoma gene in cancer WP2446 |
|  |  | CMS3 | CCLE | 59 | SNU283 LARGE INTESTINE  CL34 LARGE INTESTINE  SW1463 LARGE INTESTINE  CL40 LARGE INTESTINE |
|  |  |  | GO_BP | 421 | regulation of epithelial cell proliferation (GO:0050678)  columnar/cuboidal epithelial cell differentiation (GO:0002065)  positive regulation of epidermal growth factor receptor signaling pathway (GO:0045742) |
|  |  |  | ChEA | 257 | XRN2 22483619 ChIP-Seq HELA Human  MYC 19030024 ChIP-ChIP MESCs Mouse  MYC 28411283 ChIP-Seq MDA231-LM2-4175 Human BreastCancer  MYC 18358816 ChIP-ChIP MESCs Mouse  MYC 22102868 ChIP-Seq CA46 Human Blood BurkittsLymphoma  TTF2 22483619 ChIP-Seq HELA Human  E2F1 18555785 ChIP-Seq MESCs Mouse  EKLF 21900194 ChIP-Seq ERYTHROCYTE Mouse |
|  |  |  | WikiPathway | 55 | Metabolic reprogramming in colon cancer WP4290  Apoptosis-related network due to altered Notch3 in ovarian cancer WP2864  Senescence and Autophagy in Cancer WP615 |
|  |  | CMS4 | CCLE | 49 | CL40 LARGE INTESTINE  CL34 LARGE INTESTINE  SNU283 LARGE INTESTINE  T84 LARGE INTESTINE |
|  |  |  | GO_BP | 400 | \| SRP-dependent cotranslational protein targeting to membrane (GO:0006614) \| \| --- \| \| protein targeting to ER (GO:0045047) \| \| cotranslational protein targeting to membrane (GO:0006613) \| \| cytoplasmic translation (GO:0002181) \| \| nuclear-transcribed mRNA catabolic process \| |
|  |  |  | ChEA | 257 | \| MYC 18555785 ChIP-Seq MESCs Mouse \| \| --- \| \| MYC 19030024 ChIP-ChIP MESCs Mouse \| \| MYC 19079543 ChIP-ChIP MESCs Mouse \| \| MYC 22102868 ChIP-Seq CA46 Human Blood BurkittsLymphoma \| \| MYC 18358816 ChIP-ChIP MESCs Mouse \| \| XRN2 22483619 ChIP-Seq HELA Human \| \| MYC 28411283 ChIP-Seq MDA231-LM2-4175 Human BreastCancer \| \| E2F1 18555785 ChIP-Seq MESCs Mouse \| \| TTF2 22483619 ChIP-Seq HELA Human \| |
|  |  |  | WikiPathway | 52 | VEGFA-VEGFR2 Signaling Pathway WP3888  Senescence and Autophagy in Cancer WP615  MAPK pathway in congenital thyroid cancer WP4928 |
|  | MET2 | CMS1 | CCLE | 60 | SNU283 LARGE INTESTINE  CL34 LARGE INTESTINE  CL40 LARGE INTESTINE  LS180 LARGE INTESTINE  HT115 LARGE INTESTINE  SNU61 LARGE INTESTINE  T84 LARGE INTESTINE |
|  |  |  | GO_BP | 507 | cytoplasmic translation (GO:0002181)  SRP-dependent cotranslational protein targeting to membrane (GO:0006614)  protein targeting to ER (GO:0045047)  cotranslational protein targeting to membrane (GO:0006613) |
|  |  |  | ChEA | 283 | MYC 19079543 ChIP-ChIP MESCs Mouse  MYC 19030024 ChIP-ChIP MESCs Mouse  MYC 18555785 ChIP-Seq MESCs Mouse  MYC 18358816 ChIP-ChIP MESCs Mouse  MYC 28411283 ChIP-Seq MDA231-LM2-4175 Human BreastCancer  XRN2 22483619 ChIP-Seq HELA Human  E2F1 18555785 ChIP-Seq MESCs Mouse  MYC 22102868 ChIP-Seq CA46 Human Blood BurkittsLymphoma  TTF2 22483619 ChIP-Seq HELA Human  ZFX 18555785 ChIP-Seq MESCs Mouse |
|  |  |  | WikiPathway | 48 | Metabolic reprogramming in colon cancer WP4290  Gastric Cancer Network 2 WP2363  Apoptosis-related network due to altered Notch3 in ovarian cancer WP2864 |
|  |  | CMS2 | CCLE | 56 | SNU283 LARGE INTESTINE  CL40 LARGE INTESTINE  CL34 LARGE INTESTINE  T84 LARGE INTESTINE  HCC56 LARGE INTESTINE  SW1463 LARGE INTESTINE  NCIH508 LARGE INTESTINE |
|  |  |  | GO_BP | 472 | regulation of epithelial cell proliferation (GO:0050678)  SRP-dependent cotranslational protein targeting to membrane (GO:0006614)  cytoplasmic translation (GO:0002181)  cotranslational protein targeting to membrane (GO:0006613)  protein targeting to ER (GO:0045047)  nuclear-transcribed mRNA catabolic process  peptide biosynthetic process (GO:0043043)  translation (GO:0006412)  nuclear-transcribed mRNA catabolic process (GO:0000956)  cellular macromolecule biosynthetic process (GO:0034645) |
|  |  |  | ChEA | 308 | MYC 19079543 ChIP-ChIP MESCs Mouse  MYC 18555785 ChIP-Seq MESCs Mouse  MYC 19030024 ChIP-ChIP MESCs Mouse  MYC 18358816 ChIP-ChIP MESCs Mouse  MYC 28411283 ChIP-Seq MDA231-LM2-4175 Human BreastCancer  XRN2 22483619 ChIP-Seq HELA Human  E2F1 18555785 ChIP-Seq MESCs Mouse  MYC 22102868 ChIP-Seq CA46 Human Blood BurkittsLymphoma  TTF2 22483619 ChIP-Seq HELA Human  ZFX 18555785 ChIP-Seq MESCs Mouse  NELFA 20434984 ChIP-Seq ESCs Mouse  TAL1 20887958 ChIP-Seq HPC-7 Mouse  EKLF 21900194 ChIP-Seq ERYTHROCYTE Mouse  OCT4 18692474 ChIP-Seq MEFs Mouse |
|  |  |  | WikiPathway | 70 | Metabolic reprogramming in colon cancer WP4290  Small cell lung cancer WP4658  Bladder cancer WP2828 |
|  |  | CMS3 | CCLE | 61 | SNU283 LARGE INTESTINE  CL34 LARGE INTESTINE  SW1463 LARGE INTESTINE  CL40 LARGE INTESTINE  HT115 LARGE INTESTINE  T84 LARGE INTESTINE  SW948 LARGE INTESTINE  SNU61 LARGE INTESTINE |
|  |  |  | GO_BP | 468 | cytoplasmic translation (GO:0002181)  SRP-dependent cotranslational protein targeting to membrane (GO:0006614)  cotranslational protein targeting to membrane (GO:0006613)  protein targeting to ER (GO:0045047)  nuclear-transcribed mRNA catabolic process  peptide biosynthetic process (GO:0043043)  nuclear-transcribed mRNA catabolic process (GO:0000956)  translation (GO:0006412)  cellular macromolecule biosynthetic process (GO:0034645)  gene expression (GO:0010467)  cellular protein metabolic process (GO:0044267) |
|  |  |  | ChEA | 226 | MYC 19079543 ChIP-ChIP MESCs Mouse  MYC 18555785 ChIP-Seq MESCs Mouse  MYC 19030024 ChIP-ChIP MESCs Mouse  XRN2 22483619 ChIP-Seq HELA Human  MYC 18358816 ChIP-ChIP MESCs Mouse  MYC 28411283 ChIP-Seq MDA231-LM2-4175 Human BreastCancer  E2F1 18555785 ChIP-Seq MESCs Mouse  MYC 22102868 ChIP-Seq CA46 Human Blood BurkittsLymphoma  TTF2 22483619 ChIP-Seq HELA Human  TAL1 20887958 ChIP-Seq HPC-7 Mouse |
|  |  |  | WikiPathway | 60 | Cytoplasmic Ribosomal Proteins WP477  VEGFA-VEGFR2 Signaling Pathway WP3888  Translation Factors WP107  nsp1 from SARS-CoV-2 inhibits translation initiation in the host cell WP5027  Glycolysis in senescence WP5049  Spinal Cord Injury WP2431  Computational Model of Aerobic Glycolysis WP4629  Metabolic reprogramming in colon cancer WP4290 |
|  |  | CMS4 | CCLE | 49 | \| CL40 LARGE INTESTINE \| \| --- \| \| HCC56 LARGE INTESTINE \| \| SNU283 LARGE INTESTINE \| \| T84 LARGE INTESTINE \| \| JHOM2B OVARY \| \| CL34 LARGE INTESTINE \| \| HPAFII PANCREAS \| \| GSU STOMACH \| |
|  |  |  | GO_BP | 399 | SRP-dependent cotranslational protein targeting to membrane (GO:0006614)  cytoplasmic translation (GO:0002181)  cotranslational protein targeting to membrane (GO:0006613)  protein targeting to ER (GO:0045047)  nuclear-transcribed mRNA catabolic process  peptide biosynthetic process (GO:0043043)  nuclear-transcribed mRNA catabolic process (GO:0000956) |
|  |  |  | ChEA | 243 | \| MYC 18555785 ChIP-Seq MESCs Mouse \| \| --- \| \| MYC 19079543 ChIP-ChIP MESCs Mouse \| \| MYC 19030024 ChIP-ChIP MESCs Mouse \| \| XRN2 22483619 ChIP-Seq HELA Human \| \| MYC 18358816 ChIP-ChIP MESCs Mouse \| \| MYC 22102868 ChIP-Seq CA46 Human Blood BurkittsLymphoma \| \| MYC 28411283 ChIP-Seq MDA231-LM2-4175 Human BreastCancer \| \| TTF2 22483619 ChIP-Seq HELA Human \| \| E2F1 18555785 ChIP-Seq MESCs Mouse \| \| TAL1 20887958 ChIP-Seq HPC-7 Mouse \| |
|  |  |  | WikiPathway | 32 | Cytoplasmic Ribosomal Proteins WP477  VEGFA-VEGFR2 Signaling Pathway WP3888  Cellular Proteostasis WP4918  mRNA Processing WP411  Arachidonate Epoxygenase / Epoxide Hydrolase WP678  Parkin-Ubiquitin Proteasomal System pathway WP2359  miR-517 relationship with ARCN1 and USP1 WP3596  nsp1 from SARS-CoV-2 inhibits translation initiation in the host cell WP5027  Mitochondrial CIV Assembly WP4922  Host-pathogen interaction of human coronaviruses - MAPK signaling WP4877  Gastrin signaling pathway WP4659 |
| **KUL3** | ME | CMS1 | CCLE | 77 | CL40 LARGE INTESTINE  SNU283 LARGE INTESTINE  GSU STOMACH  SNU61 LARGE INTESTINE  SNU245 BILIARY TRACT  CL34 LARGE INTESTINE  HT29 LARGE INTESTINE  HT115 LARGE INTESTINE  T84 LARGE INTESTINE  ASPC1 PANCREAS  JHOM2B OVARY |
|  |  |  | GO_BP | 433 | cotranslational protein targeting to membrane (GO:0006613)  SRP-dependent cotranslational protein targeting to membrane (GO:0006614)  cytoplasmic translation (GO:0002181)  protein targeting to ER (GO:0045047)  nuclear-transcribed mRNA catabolic process  peptide biosynthetic process (GO:0043043)  nuclear-transcribed mRNA catabolic process (GO:0000956)  translation (GO:0006412) |
|  |  |  | ChEA | 262 | MYC 19079543 ChIP-ChIP MESCs Mouse  XRN2 22483619 ChIP-Seq HELA Human  MYC 18555785 ChIP-Seq MESCs Mouse  MYC 19030024 ChIP-ChIP MESCs Mouse  MYC 18358816 ChIP-ChIP MESCs Mouse  E2F1 18555785 ChIP-Seq MESCs Mouse  TTF2 22483619 ChIP-Seq HELA Human  EKLF 21900194 ChIP-Seq ERYTHROCYTE Mouse  MYC 28411283 ChIP-Seq MDA231-LM2-4175 Human BreastCancer |
|  |  |  | WikiPathway | 94 | Cytoplasmic Ribosomal Proteins WP477  VEGFA-VEGFR2 Signaling Pathway WP3888  Gastrin signaling pathway WP4659  Zinc homeostasis WP3529  PPAR signaling pathway WP3942  Senescence and Autophagy in Cancer WP615  Vitamin D Receptor Pathway WP2877  IL-18 signaling pathway WP4754  Hepatitis C and Hepatocellular Carcinoma WP3646  Copper homeostasis WP3286  Host-pathogen interaction of human coronaviruses - MAPK signaling WP4877  Amino Acid metabolism WP3925  Photodynamic therapy-induced AP-1 survival signaling. WP3611 |
|  |  | CMS2 | CCLE | 58 | SNU283 LARGE INTESTINE  CL40 LARGE INTESTINE  HT115 LARGE INTESTINE  CL34 LARGE INTESTINE  SNU61 LARGE INTESTINE  NCIH508 LARGE INTESTINE  GSU STOMACH  CW2 LARGE INTESTINE  T84 LARGE INTESTINE  SW1463 LARGE INTESTINE  SNU1040 LARGE INTESTINE  CL14 LARGE INTESTINE |
|  |  |  | GO_BP | 461 | SRP-dependent cotranslational protein targeting to membrane (GO:0006614)  cytoplasmic translation (GO:0002181)  cotranslational protein targeting to membrane (GO:0006613)  protein targeting to ER (GO:0045047)  nuclear-transcribed mRNA catabolic process  nuclear-transcribed mRNA catabolic process (GO:0000956)  peptide biosynthetic process (GO:0043043)  translation (GO:0006412)  cellular macromolecule biosynthetic process (GO:0034645)  rRNA processing (GO:0006364)  rRNA metabolic process (GO:0016072)  ribosome biogenesis (GO:0042254)  gene expression (GO:0010467) |
|  |  |  | ChEA | 322 | MYC 19079543 ChIP-ChIP MESCs Mouse  MYC 18555785 ChIP-Seq MESCs Mouse  XRN2 22483619 ChIP-Seq HELA Human  MYC 19030024 ChIP-ChIP MESCs Mouse  MYC 18358816 ChIP-ChIP MESCs Mouse  E2F1 18555785 ChIP-Seq MESCs Mouse  MYC 28411283 ChIP-Seq MDA231-LM2-4175 Human BreastCancer  TTF2 22483619 ChIP-Seq HELA Human  MYC 22102868 ChIP-Seq CA46 Human Blood BurkittsLymphoma  TAL1 20887958 ChIP-Seq HPC-7 Mouse  NELFA 20434984 ChIP-Seq ESCs Mouse  EKLF 21900194 ChIP-Seq ERYTHROCYTE Mouse  OCT4 18692474 ChIP-Seq MEFs Mouse  KLF4 19030024 ChIP-ChIP MESCs Mouse  ZFX 18555785 ChIP-Seq MESCs Mouse |
|  |  |  | WikiPathway | 72 | Cytoplasmic Ribosomal Proteins WP477  VEGFA-VEGFR2 Signaling Pathway WP3888  Zinc homeostasis WP3529  miR-517 relationship with ARCN1 and USP1 WP3596  Translation Factors WP107  Copper homeostasis WP3286  nsp1 from SARS-CoV-2 inhibits translation initiation in the host cell WP5027  Vitamin D Receptor Pathway WP2877  Photodynamic therapy-induced AP-1 survival signaling. WP3611  Host-pathogen interaction of human coronaviruses - MAPK signaling WP4877  Nuclear Receptors Meta-Pathway WP2882  mRNA Processing WP411  Pre-implantation embryo WP3527 |
|  |  | CMS3 | CCLE | 81 | CL40 LARGE INTESTINE  SNU283 LARGE INTESTINE  CL34 LARGE INTESTINE  GSU STOMACH  T84 LARGE INTESTINE  LS180 LARGE INTESTINE  SNU245 BILIARY TRACT  HT115 LARGE INTESTINE  SW1463 LARGE INTESTINE  SNU1040 LARGE INTESTINE |
|  |  |  | GO_BP | 397 | SRP-dependent cotranslational protein targeting to membrane (GO:0006614)  protein targeting to ER (GO:0045047)  cotranslational protein targeting to membrane (GO:0006613)  cytoplasmic translation (GO:0002181)  nuclear-transcribed mRNA catabolic process  peptide biosynthetic process (GO:0043043)  nuclear-transcribed mRNA catabolic process (GO:0000956)  translation (GO:0006412)  cellular macromolecule biosynthetic process (GO:0034645)  gene expression (GO:0010467)  cellular protein metabolic process (GO:0044267) |
|  |  |  | ChEA | 223 | MYC 19079543 ChIP-ChIP MESCs Mouse  MYC 18555785 ChIP-Seq MESCs Mouse  XRN2 22483619 ChIP-Seq HELA Human  MYC 19030024 ChIP-ChIP MESCs Mouse  E2F1 18555785 ChIP-Seq MESCs Mouse  MYC 18358816 ChIP-ChIP MESCs Mouse  TTF2 22483619 ChIP-Seq HELA Human  MYC 28411283 ChIP-Seq MDA231-LM2-4175 Human BreastCancer  MYC 22102868 ChIP-Seq CA46 Human Blood BurkittsLymphoma  OCT4 18692474 ChIP-Seq MEFs Mouse  NELFA 20434984 ChIP-Seq ESCs Mouse  ZFX 18555785 ChIP-Seq MESCs Mouse  EKLF 21900194 ChIP-Seq ERYTHROCYTE Mouse |
|  |  |  | WikiPathway | 59 | Cytoplasmic Ribosomal Proteins WP477  Zinc homeostasis WP3529  VEGFA-VEGFR2 Signaling Pathway WP3888  PPAR signaling pathway WP3942  Copper homeostasis WP3286  miR-517 relationship with ARCN1 and USP1 WP3596  Vitamin D Receptor Pathway WP2877  Nuclear Receptors Meta-Pathway WP2882  Gastrin signaling pathway WP4659  Amino Acid metabolism WP3925  Photodynamic therapy-induced AP-1 survival signaling. WP3611  ID signaling pathway WP53  Mitochondrial complex III assembly WP4921 |
|  |  | CMS4 | CCLE | 66 | CL40 LARGE INTESTINE  SNU283 LARGE INTESTINE  GSU STOMACH  SNU61 LARGE INTESTINE  HT115 LARGE INTESTINE  T84 LARGE INTESTINE  CL34 LARGE INTESTINE  SNUC4 LARGE INTESTINE  CW2 LARGE INTESTINE  SNU245 BILIARY TRACT  NCIH508 LARGE INTESTINE  SNU1040 LARGE INTESTINE  SW1463 LARGE INTESTINE |
|  |  |  | GO_BP | 486 | SRP-dependent cotranslational protein targeting to membrane (GO:0006614)  cotranslational protein targeting to membrane (GO:0006613)  cytoplasmic translation (GO:0002181)  protein targeting to ER (GO:0045047)  nuclear-transcribed mRNA catabolic process  peptide biosynthetic process (GO:0043043)  nuclear-transcribed mRNA catabolic process (GO:0000956)  translation (GO:0006412)  cellular macromolecule biosynthetic process (GO:0034645)  rRNA processing (GO:0006364)  rRNA metabolic process (GO:0016072)  ribosome biogenesis (GO:0042254)  cellular protein metabolic process (GO:0044267) |
|  |  |  | ChEA | 269 | MYC 19079543 ChIP-ChIP MESCs Mouse  XRN2 22483619 ChIP-Seq HELA Human  MYC 18555785 ChIP-Seq MESCs Mouse  MYC 19030024 ChIP-ChIP MESCs Mouse  E2F1 18555785 ChIP-Seq MESCs Mouse  TTF2 22483619 ChIP-Seq HELA Human  MYC 18358816 ChIP-ChIP MESCs Mouse  NELFA 20434984 ChIP-Seq ESCs Mouse  CEBPB 24764292 ChIP-Seq MC3T3 Mouse  EKLF 21900194 ChIP-Seq ERYTHROCYTE Mouse  MYC 28411283 ChIP-Seq MDA231-LM2-4175 Human BreastCancer  OCT4 18692474 ChIP-Seq MEFs Mouse |
|  |  |  | WikiPathway | 100 | \| Cytoplasmic Ribosomal Proteins WP477 \| \| --- \| \| Nonalcoholic fatty liver disease WP4396 \| \| Electron Transport Chain (OXPHOS system in mitochondria) WP111 \| \| VEGFA-VEGFR2 Signaling Pathway WP3888 \| \| IL-18 signaling pathway WP4754 \| \| Mitochondrial complex III assembly WP4921 \| \| Mitochondrial CIV Assembly WP4922 \| \| Zinc homeostasis WP3529 \| \| Myometrial relaxation and contraction pathways WP289 \| \| Spinal Cord Injury WP2431 \| \| PPAR signaling pathway WP3942 \| \| Senescence and Autophagy in Cancer WP615 \| |

Table_S 4: List of effective genes common in disease and drug signatures. The pattern of expression of these genes in disease is the opposite of their expression in drug treatment

| **UP**  **(Down in disease & UP by drug)** | **Down**  **(UP in disease & Down by drug)** | **DRUG** |
| --- | --- | --- |
| SFN, IER3, ALDOA | IGFBP3, S100A13, MMP1, CDKN2A, MYL9, SOX4, FKBP4, DUSP4, GLRX, UBE2L6, NFATC4, NUCB2 | Tezacaftor |
| ACAA1 | HSPD1, S100A13, MMP1, RPS6, UFM1, DNAJB1, KDELR2, POLR2K, BAG3, CYCS, VPS72, DSG2, TRIB1, LYPLA1, TSPAN6, REEP5 | Famciclovir |
| CDKN1A, FOS, HADH, BAD | STMN1, BMP4, PCBD1, UBE2C, MLEC, SOX4, HSPA8, GAPDH, GTF2A2, DDIT4, KDELR2, EIF4EBP1, MTHFD2, POLR2K, PGAM1, FHL2, BAG3, RHOA, MRPL12, GNAS, MFSD10, INTS3, RAB27A, PRKAG2, TRAP1, TRIB1, VDAC1, REEP5 | Fenticonazole |

Table S 5: Details of the selected drugs obtained from the iLINCS website.

| **State** | **MET1**  **CMS1** | **MET1**  **CMS2** | **MET1**  **CMS3** | **MET1**  **CMS4** | **MET2**  **CMS1** | **MET2**  **CMS2** | **MET2**  **CMS3** | **MET2**  **CMS4** | **ME**  **CMS1** | **ME**  **CMS2** | **ME**  **CMS3** | **ME**  **CMS4** |
| --- | --- | --- | --- | --- | --- | --- | --- | --- | --- | --- | --- | --- |
| PerturbagenId | BRD-K45033733 | BRD-K45033733 | BRD-K45033733 | BRD-K45033733 | BRD-K45033733 | BRD-K45033733 | BRD-K45033733 | BRD-K45033733 | BRD-K45033733 | BRD-K45033733 | BRD-K45033733 | - |
| Perturbagen | Famciclovir | Famciclovir | Famciclovir | Famciclovir | Famciclovir | Famciclovir | Famciclovir | Famciclovir | Famciclovir | Famciclovir | Famciclovir | - |
| GeneTargets |  |  |  |  |  |  |  |  |  |  |  | - |
| Correlation | - | - | - | - | - | - | - | - | - | - | - | - |
| NoOfSignatures | 33 | 33 | 33 | 33 | 33 | 33 | 33 | 33 | 33 | 33 | 33 | - |
| pValue | 2.26E-15 | 4.65E-09 | 5.65E-28 | 1.02E-07 | 7.76E-15 | 1.15E-09 | 6.97E-20 | 0.0017244 | 0.00296126 | 1.94E-21 | 0.0113331 | - |
| zScore | 7.8394100 | 5.7429126 | 10.901791 | 5.1960923 | 7.6831960 | 5.9752257 | 9.0526993 | 2.9246049 | 2.7520407 | 9.4355898 | 2.2790117 | - |
|  |  |  |  |  |  |  |  |  |  |  |  |  |
| PerturbagenId | - | - | - | BRD-A33697453 | - | - | - | - | - | BRD-A33697453 | - | - |
| Perturbagen | - | - | - | Fenticonazole Nitrate | - | - | - | - | - | Fenticonazole Nitrate | - | - |
| GeneTargets | - | - | - | NA | - | - | - | - | - | NA | - | - |
| Correlation | - | - | - | - | - | - | - | - | - | - | - | - |
| NoOfSignatures | - |  | - | 26 | - | - | - | - | - | 26 | - | - |
| pValue | - | - | - | 8.47492E-06 | - | - | - | - | - | 4.23E-08 | - | - |
| zScore | - | - | - | 4.301692454 | - | - | - | - | - | 5.3572375 | - | - |
|  |  |  |  |  |  |  |  |  |  |  |  |  |
| GeneTarget | - | - | - | CFTR | CFTR | CFTR | CFTR | CFTR | CFTR | CFTR | CFTR | CFTR |
| Pathways | - | - | - | ABC transporters | ABC transporter | ABC transporter | ABC transporter | ABC transporter | ABC transporters | ABC transporters | ABC transporters | ABC transporters |
| Correlation | - | - | - | - | - | - | - | - | - | - | - | - |
| NoOfSignatures | - | - | - | 9 | 9 | 9 | 9 | 9 | 9 | 9 | 9 | 9 |
| pValue | - | - | - | 0.001206256 | 0.0113976 | 0.0042324 | 0.0010740 | 3.901E-06 | 3.51E-14 | 1.72E-64 | 4.11E-07 | 6.71E-21 |
| zScore | - | - | - | 3.034104073 | 2.2768494 | 2.6329380 | 3.0689618 | 4.4705909 | 7.48737361 | 16.9157572 | 4.93002549 | 9.30491138 |
|  |  |  |  |  |  |  |  |  |  |  |  |  |
| GeneTarget | - | - | ACLY | ACLY | - | - | ACLY | ACLY | ACLY | ACLY | ACLY | ACLY |
| Pathways | - | - | TCA cycle | TCA cycle | - | - | TCA cycle | TCA cycle | TCA cycle | TCA cycle | TCA cycle | TCA cycle |
| Correlation | - | - | - | - | - | - | - | - | - | - | - | - |
| NoOfSignatures | - | - | 25 | 25 | - | - | 25 | 25 | 25 | 25 | 25 | 25 |
| pValue | - | - | 0.0002843 | 1.50E-12 | - | - | 0.0019086 | 8.45E-39 | 2.78E-12 | 0.00604164 | 6.40E-20 | 1.75E-07 |
| zScore | - | - | 3.4461508 | 6.977717786 | - | - | 2.8928839 | 12.975250 | 6.89058081 | 2.50970301 | 9.06204589 | 5.09386542 |

Table S 6: Details of similar drugs using iLINCS website.

| Candidate drug | iLINCS signature | Common chemical compounds | Approved drugs |
| --- | --- | --- | --- |
| Trifluridine and Tipiracil Hydrochloride | HT29_24h_0.12uM  HT29_24h_1.11uM | 359 | Fenticonazole Nitrate  Diphenylpyraline  Tezampanel  BMY-14802 |
| Irinotecan Hydrochloride | HT29_6h_10uM  HT29_24h_3.3uM  HT29_24h_10uM | 60 | Diphenylpyraline |
| Regorafenib | HT29_24h_0.04uM  HT29_24h_0.12uM  HT29_24h_0.37uM  HT29_24h_1.11uM  HT29_24h_3.33uM  HT29_24h_10uM | 3 | Fenticonazole Nitrate  Diphenylpyraline |
| Floxuridine | HT29_6h_10uM  HT29_24h_0.04uM  HT29_24h_0.12uM  HT29_24h_0.37uM  HT29_24h_1.11uM  HT29_24h_3.33uM  HT29_24h_10uM | 29 | - |
| Fluorouracil Injection | HT29_6h_10uM  HT29_24h_10uM | 163 | - |
| Bevacizumab | - | - | - |
| Capecitabine | - | - | - |
| Cetuximab | - | - | - |
| Ramucirumab | - | - | - |
| Oxaliplatin | - | - | - |
| Fruquintinib | - | - | - |
| Ipilimumab | - | - | - |
| Pembrolizumab | - | - | - |
| Leucovorin Calcium | - | - | - |
| Nivolumab | - | - | - |
| Panitumumab | - | - | - |
| Tucatinib | - | - | - |
| Ziv-Aflibercept | - | - | - |

Table S 7: Selected drugs and their details in concentration, time, cell line, tissue, gene signature (top 10) and compounds related to them (top 10). In additional file 2, Excel files related to all gene signatures and related compounds of 4 selected drugs are available. All the data in this table are extracted from the iLINCS website.

| Drug (Perturbagen) | Cell Line | Tissue | Time | Concentration | Signature (top 10) | | Related compounds (top 10) |
| --- | --- | --- | --- | --- | --- | --- | --- |
|  |  |  |  |  | Up (gene name/ LFC) | Down (gene name/ LFC) |  |
| Famciclovir | HT29 | large intestine | 24h | 10uM | \| SCRN1 \| 6.76296 \| \| --- \| --- \| \| CASP10 \| 6.35773 \| \| AGL \| 2.20888 \| \| FSD1 \| 2.01329 \| \| ITGB5 \| 2.00521 \| \| PIK3C2B \| 1.9314 \| \| STK25 \| 1.84621 \| \| CIAPIN1 \| 1.77155 \| \| RAP1GAP \| 1.72794 \| \| PWP1 \| 1.72188 \| | \| PECR \| -1.43462 \| \| --- \| --- \| \| CDKN2A \| -1.49036 \| \| GOLT1B \| -1.52104 \| \| GADD45A \| -1.56026 \| \| CD58 \| -1.69751 \| \| TSPAN6 \| -1.71325 \| \| BAX \| -1.75941 \| \| CLTC \| -2.19841 \| \| PSME1 \| -2.39698 \| \| FASTKD5 \| -3.53228 \| | Famciclovir  Proglumide  Ticagrelor  Zolpidem  Sarsasapogenin  tetrahydropalmatine  TAK-285  Taselisib  Pimavanserin  Mubritinib (TAK 165) |
|  | HT29 | large intestine | 24h | 3.33uM | \| PPP1R13B \| 7.60554 \| \| --- \| --- \| \| GNA15 \| 6.47064 \| \| GPATCH8 \| 4.65105 \| \| STXBP1 \| 3.4709 \| \| TP53BP1 \| 2.71639 \| \| TBC1D31 \| 2.52807 \| \| CSK \| 2.13916 \| \| MAST2 \| 2.12691 \| \| DDIT4 \| 2.05321 \| \| STK25 \| 2.04466 \| | \| ITGB1BP1 \| -1.60165 \| \| --- \| --- \| \| PSME1 \| -1.66469 \| \| CTNNAL1 \| -1.74908 \| \| GOLT1B \| -1.78026 \| \| TXNDC9 \| -1.78843 \| \| CRYZ \| -2.03027 \| \| TMED10 \| -2.05966 \| \| NFKB2 \| -2.08737 \| \| EVL \| -2.12855 \| \| HSPD1 \| -3.23418 \| | \| Famciclovir \| \| --- \| \| Ticagrelor \| \| Proglumide \| \| tetrahydropalmatine \| \| Pimavanserin \| \| AZD1981 \| \| Temoporfin \| \| Lenvatinib (E7080) \| \| Zolpidem \| \| FK888 \| |
|  | HT29 | large intestine | 24h | 1.11uM | \| MYCBP2 \| 1.73095 \| \| --- \| --- \| \| ARHGAP1 \| 1.77355 \| \| TARBP1 \| 1.79945 \| \| TSEN2 \| 1.803 \| \| CCL2 \| 1.83945 \| \| RBM6 \| 2.0139 \| \| NUP133 \| 2.03305 \| \| AKAP8 \| 2.03415 \| \| ICAM1 \| 7.3921 \| \| GNA15 \| 10 \| | \| PRKCQ \| -4.0986 \| \| --- \| --- \| \| BAG3 \| -2.6612 \| \| ARPP19 \| -2.23755 \| \| MMP1 \| -2.1414 \| \| HAT1 \| -2.13805 \| \| GOLT1B \| -2.06635 \| \| RPA3 \| -2.0111 \| \| LIPA \| -1.97255 \| \| ATP6V1D \| -1.91045 \| \| DCK \| -1.89995 \| | \| Famciclovir \| \| --- \| \| Ifosfamide \| \| Proglumide \| \| Ticagrelor \| \| GSK 1059615 \| \| tetrahydropalmatine \| \| Ergothioneine \| \| Taselisib \| \| C23H24O8 \| \| Pimavanserin \| |
|  | HT29 | large intestine | 24h | 0.04uM | \| LRRC16A \| 6.03267 \| \| --- \| --- \| \| EPHA3 \| 3.1959 \| \| RASA1 \| 2.91583 \| \| ARHGEF2 \| 2.7363 \| \| KEAP1 \| 2.53795 \| \| ABL1 \| 2.48337 \| \| IGF2R \| 2.33282 \| \| DNM1 \| 2.1922 \| \| GPATCH8 \| 2.14656 \| \| CDC45 \| 2.13409 \| | \| MYLK \| -3.61119 \| \| --- \| --- \| \| RPS6 \| -3.06543 \| \| DCK \| -2.92694 \| \| EIF5 \| -2.8292 \| \| GOLT1B \| -2.69378 \| \| GMNN \| -2.55005 \| \| HSPD1 \| -2.44978 \| \| MOK \| -2.34299 \| \| TXNDC9 \| -2.25538 \| \| HACD3 \| -2.07645 \| | Famciclovir  Ticagrelor  Sarsasapogenin  Pimavanserin  Proglumide  tetrahydropalmatine  Ifosfamide  Zolpidem  Temoporfin  Lafutidine |
| Tezacaftor | HT29 | large intestine | 24h | 10uM | \| HMOX1 \| 4.11674 \| \| --- \| --- \| \| MOK \| 3.02335 \| \| ELOVL6 \| 2.93075 \| \| CDK6 \| 2.55865 \| \| DDIT4 \| 2.1464 \| \| RRP1B \| 2.12183 \| \| MYC \| 2.08709 \| \| MRPL12 \| 2.03975 \| \| CEBPA \| 1.9868 \| \| HDAC2 \| 1.80157 \| | \| CDKN2A \| -7.03287 \| \| --- \| --- \| \| SOX4 \| -3.58236 \| \| IQGAP1 \| -2.86147 \| \| GLRX \| -2.82104 \| \| PRSS23 \| -2.16418 \| \| PPP2R5E \| -2.12184 \| \| S100A13 \| -1.91294 \| \| RGS2 \| -1.76698 \| \| HMGCS1 \| -1.74183 \| \| SPDEF \| -1.70462 \| | Tezacaftor  Pioglitazone  Rosiglitazone  GSK-3 Inhibitor IX  BRD-K00544996  Troglitazone  Inolitazone (dihydrochloride)  Narlaprevir  Ulipristal |
|  | HT29 | large intestine | 24h | 3.33uM | \| C2CD5 \| 9.99809 \| \| --- \| --- \| \| CCNA1 \| 6.12003 \| \| HMOX1 \| 3.39506 \| \| ELOVL6 \| 2.81919 \| \| BLMH \| 2.39343 \| \| CDH3 \| 1.92717 \| \| DDX10 \| 1.90424 \| \| PRKCD \| 1.76076 \| \| MYC \| 1.75768 \| \| TMEM109 \| 1.64679 \| | \| CANT1 \| -6.97682 \| \| --- \| --- \| \| ERBB2 \| -6.41999 \| \| SOX4 \| -3.13674 \| \| GRN \| -2.97865 \| \| GLRX \| -2.45516 \| \| APP \| -2.3496 \| \| RGS2 \| -2.20736 \| \| ADGRG1 \| -1.8329 \| \| KLHL9 \| -1.83029 \| \| CDKN1B \| -1.70005 \| | Tezacaftor  Rosiglitazone  Asenapine maleate  FPL-55712  Pioglitazone  Pyrilamine  LY2811376  CANAGLIFLOZIN  NKP608  Etanidazole |
|  | HT29 | large intestine | 24h | 1.11uM | \| SSBP2 \| 7.77484 \| \| --- \| --- \| \| ABCC5 \| 7.11052 \| \| RAB21 \| 4.0339 \| \| MAST2 \| 3.2255 \| \| DNAJB2 \| 2.94333 \| \| ELOVL6 \| 2.32293 \| \| TRAP1 \| 1.96192 \| \| COG2 \| 1.77702 \| \| ICAM3 \| 1.665 \| \| PYCR1 \| 1.6174 \| | \| CDKN2A \| -6.56273 \| \| --- \| --- \| \| IQGAP1 \| -1.98801 \| \| ITFG1 \| -1.83179 \| \| UBE2L6 \| -1.59494 \| \| PRSS23 \| -1.50522 \| \| BLVRA \| -1.5024 \| \| IGF2R \| -1.49912 \| \| GADD45A \| -1.49872 \| \| WASF3 \| -1.49155 \| \| DYNLT3 \| -1.4776 \| | Tezacaftor  Pyrilamine  Asenapine maleate  FPL-55712  Etanidazole  Diosmin  LY2811376  Ribavirin  Suvorexant (MK-4305)  MK-0974 |
|  | HT29 | large intestine | 24h | 0.12uM | \| ADGRE5 \| 9.17484 \| \| --- \| --- \| \| PRKCH \| 6.41546 \| \| USP7 \| 5.56003 \| \| MOK \| 3.9701 \| \| SLC37A4 \| 2.63424 \| \| GALE \| 2.47751 \| \| RALGDS \| 1.95795 \| \| EIF4EBP1 \| 1.90848 \| \| PRR7 \| 1.86928 \| \| PRAF2 \| 1.71527 \| | \| MBNL2 \| -6.65967 \| \| --- \| --- \| \| LAP3 \| -6.01992 \| \| IQGAP1 \| -2.3301 \| \| PPP2R5E \| -2.20842 \| \| GPER1 \| -1.98598 \| \| APP \| -1.87714 \| \| GLRX \| -1.69069 \| \| RAI14 \| -1.61363 \| \| CEP57 \| -1.49595 \| \| PPOX \| -1.43769 \| | \| Tezacaftor \| \| --- \| \| Asenapine maleate \| \| FPL-55712 \| \| Pyrilamine \| \| SB525334 \| \| Imatinib \| \| BMS-777607 \| \| MLS001065847 \| \| GSK-3 Inhibitor II \| |
| Target Gene_ACLY  (bempedoic acid) | HT29 | large intestine | 96h | 1 Ul | \| HMGCR \| 4.55044 \| \| --- \| --- \| \| HMGCS1 \| 4.36318 \| \| NSDHL \| 4.23853 \| \| TMEM97 \| 3.75982 \| \| ACAT2 \| 3.53994 \| \| EBP \| 3.46983 \| \| SLC2A6 \| 3.07521 \| \| INSIG1 \| 3.02092 \| \| STXBP1 \| 2.84933 \| \| ELOVL6 \| 2.68006 \| | \| ACLY \| -5.08995 \| \| --- \| --- \| \| HIST1H2BK \| -3.82479 \| \| TMEM109 \| -2.69389 \| \| TNIP1 \| -2.23561 \| \| MLEC \| -2.0461 \| \| ZW10 \| -1.85658 \| \| BDH1 \| -1.72292 \| \| LYPLA1 \| -1.60622 \| \| HSPB1 \| -1.51922 \| \| ARPP19 \| -1.48007 \| | MG-132  179324-69-7  Thioridazine  Apilimod  CEPHARANTHINE  Fluspirilene  Orlistat  E6 Berbamine  MLS000095184  SCHEMBL2394914 |
| Fenticonazole Nitrate | HT29 | large intestine | 24h | 0.04uM | \| NFKB2 \| 7.35203 \| \| --- \| --- \| \| PIK3R3 \| 7.34384 \| \| SIRT3 \| 7.24206 \| \| E2F2 \| 7.17337 \| \| CXCL2 \| 6.57493 \| \| MMP1 \| 6.49801 \| \| PRKCQ \| 6.3728 \| \| IKZF1 \| 6.26343 \| \| GNAI1 \| 6.17798 \| \| TUBB6 \| 5.93451 \| | \| ITGAE \| -6.27663 \| \| --- \| --- \| \| PARP2 \| -5.28662 \| \| ACOT9 \| -5.15615 \| \| IARS2 \| -4.80754 \| \| CHMP4A \| -4.73289 \| \| GAPDH \| -4.15061 \| \| MACF1 \| -3.86884 \| \| GALE \| -3.56875 \| \| CASC3 \| -3.21465 \| \| PDIA5 \| -3.19865 \| | \| CGP 60474 \| \| --- \| \| Fenticonazole Nitrate \| \| Dinaciclib \| \| Staurosporine \| \| 179324-69-7 \| \| OTSSP167 \| \| Mitoxantrone \| \| Alvocidib \| \| Sulpiride \| \| Cobicistat(GS9350) \| |
|  | HT29 | large intestine | 24h | 0.12uM | \| PIK3R3 \| 4.946 \| \| --- \| --- \| \| SPP1 \| 4.45597 \| \| MAPKAPK3 \| 3.30457 \| \| RNH1 \| 2.89429 \| \| DPH2 \| 2.78091 \| \| MEST \| 2.64092 \| \| PAN2 \| 2.54177 \| \| PPP2R5E \| 2.5051 \| \| NPDC1 \| 2.45984 \| \| KIF1BP \| 2.40025 \| | \| HADH \| -2.92569 \| \| --- \| --- \| \| EZH2 \| -2.53942 \| \| ACOT9 \| -2.38225 \| \| ARFIP2 \| -2.21109 \| \| ATP6V1D \| -2.09018 \| \| PNP \| -2.06294 \| \| ECD \| -2.05939 \| \| MYO10 \| -2.01996 \| \| RPA1 \| -2.01765 \| \| EPRS \| -2.0017 \| | \| 179324-69-7 \| \| --- \| \| CGP 60474 \| \| Fenticonazole Nitrate \| \| Dinaciclib \| \| MG-132 \| \| Diphenylpyraline \| \| Sulpiride \| \| Defactinib \| \| Taranabant \| \| OTSSP167 \| |
|  | HT29 | large intestine | 24h | 0.37uM | \| NFKB2 \| 6.82321 \| \| --- \| --- \| \| NFATC4 \| 5.92389 \| \| IDE \| 4.61538 \| \| CHIC2 \| 3.39641 \| \| INSIG1 \| 2.57374 \| \| TOR1A \| 2.19913 \| \| FAS \| 1.9276 \| \| HMGCR \| 1.88598 \| \| GPER1 \| 1.79406 \| \| SOCS2 \| 1.69097 \| | \| HSPA8 \| -5.83381 \| \| --- \| --- \| \| ITGAE \| -3.8469 \| \| VAT1 \| -2.37498 \| \| LAMA3 \| -1.89919 \| \| MFSD10 \| -1.74734 \| \| GNAS \| -1.73893 \| \| CASC3 \| -1.6848 \| \| TERF2IP \| -1.68118 \| \| ETFB \| -1.61923 \| \| TIMM17B \| -1.55213 \| | \| CGP 60474 \| \| --- \| \| Fenticonazole Nitrate \| \| Dinaciclib \| \| Alvocidib \| \| Diphenylpyraline \| \| Mitoxantrone \| \| Cobicistat (GS-9350) \| \| BMY-14802 \| \| Sulpiride \| \| Taranabant \| |
|  | HT29 | large intestine | 24h | 10uM | \| GFOD1 \| 5.10465 \| \| --- \| --- \| \| ORC1 \| 4.85355 \| \| SQRDL \| 4.41739 \| \| INSIG1 \| 3.24001 \| \| TSC22D3 \| 2.9255 \| \| ELOVL6 \| 2.9021 \| \| PHGDH \| 2.84669 \| \| TIPARP \| 2.6502 \| \| CLIC4 \| 2.61914 \| \| BAMBI \| 2.21094 \| | \| DNTTIP2 \| -5.60135 \| \| --- \| --- \| \| TCTA \| -2.53417 \| \| MFSD10 \| -2.34701 \| \| FASTKD5 \| -2.24738 \| \| KDM3A \| -2.24176 \| \| SOX4 \| -2.16333 \| \| BIRC5 \| -2.15093 \| \| RNMT \| -2.14211 \| \| REEP5 \| -2.11663 \| \| STMN1 \| -2.11647 \| | \| CGP 60474 \| \| --- \| \| Fenticonazole Nitrate \| \| Dinaciclib \| \| 179324-69-7 \| \| Staurosporine \| \| Dexniguldipine \| \| Diphenylpyraline \| \| Neratinib \| \| ABT-333 \| \| MG-132 \| |
