# Supplementary material for: Single-cell transcriptional signature-based drug repurposing and in vitro evaluation in colorectal cancer
Source: BMC Cancer. 2024 Mar 25;24:371. doi: 10.1186/s12885-024-12142-8 (PMC10962075; doi:10.1186/s12885-024-12142-8)
Supplement: Supplementary file 2 — Supplementary Material 2 [file 12885_2024_12142_MOESM2_ESM.docx]

Note: The supplementary information files are available in the google drive repository,

[**https://drive.google.com/drive/folders/1HS7zyLG-WgIGT6P8NhcQP55tAJkLQbSu?usp=sharing**](https://drive.google.com/drive/folders/1HS7zyLG-WgIGT6P8NhcQP55tAJkLQbSu?usp=sharing)

**Single-cell transcriptional signature-based drug repurposing and in vitro evaluation in colorectal cancer**

**Roohallah Mahdi-Esferizi^1^, Zahra Shiasi^1^,** **Razieh Heidari^1^, Ali Najafi^2^, Issa Mahmoudi^3^, Fatemeh Elahian^1^, and Shahram Tahmasebian^4,^***

^1^ Department of Medical Biotechnology, School of Advanced Technologies, Shahrekord University of Medical Sciences, Shahrekord, Iran.

^2^ Molecular Biology Research Center, Systems Biology and Poisonings Institute, Baqiyatallah University of Medical Sciences, Tehran, Iran.

^3^ Information Technology Department, Shahrekord University of Medical Sciences, Shahrekord, Iran.

^4^ Cellular and Molecular Research Center, Basic Health Sciences Institute, Shahrekord University of Medical Sciences, Shahrekord, Iran.

* Corresponding author

**Shahram Tahmasebian**

Cellular and Molecular Research Center, Basic Health Sciences Institute, Shahrekord University of Medical Sciences, Shahrekord, Iran ([stahmasebian@gmail.com](mailto:stahmasebian@gmail.com))

**This includes:**

**SUPPLEMENTARY INFORMATION PART 1:**

The files of DEGs and Enrichment results. After carrying out DEGs analysis by ASAP, the results are in Excel files. four types of enrichment analyses were performed, which are: (1) Gene ontology (GO)_biological process. (2) Cancer Cell Line Encyclopedia (CCLE). (3) WikiPathway. (4) ChIP Enrichment Analysis (ChEA). These results are shown for each state separately, in different Excel files.

**SUPPLEMENTARY INFORMATION PART 2:**

The files of connected LINCS chemical perturbagens and connected LINCS gene knockdowns for each dataset were obtained from the iLINCS website.

**SUPPLEMENTARY INFORMATION PART 3:**

The files of connected LINCS chemical perturbagens and connected LINCS gene knockdowns for each drug in different times and different concentrations were obtained from the iLINCS website.
